# Cracking the shield: oncolytic viruses versus the tumor-immune fortress

**DOI:** 10.1186/s12935-026-04331-1

**Published:** 2026-05-25

**Authors:** Azza M. El-Derby, Nouran T. Salem, Noha Hesham, Tamer Z. Salem

**Affiliations:** 1 Biomedical Sciences Program, UST, Zewail City of Science and Technology, October Gardens, 6th of October City, Giza, 12578 Egypt; 2 Molecular Biology and Virology Laboratory (MBVL), Zewail City of Science and Technology, October Gardens, 6th of October City, Giza, 12578 Egypt; 3 Center for X-ray Determination of the Structure of Matter (CXDS), Zewail City of Science and Technology, October Gradens, 6th of October City, 12578 Giza, Egypt

**Keywords:** Oncolytic Viruses (OVs), Tumor Microenvironment (TME), Hepatocellular Carcinoma (HCC), Viral engineering, Immunotherapy

## Abstract

Oncolytic viruses (OVs) have emerged as a promising cancer therapy due to their natural selectivity to replicate in and destroy cancer cells. However, despite encouraging preclinical and early clinical outcomes, the therapeutic efficacy of OVs in solid tumors remains limited. Recently, OVs have been genetically modified to enhance tumor specificity, promote antitumor immune activation, and overcome barriers imposed by the tumor microenvironment (TME). Hepatocellular carcinoma presents a uniquely challenging solid tumor microenvironment for oncolytic virotherapy, characterized by dense fibrotic stroma, potent hepatic immune clearance, and immunosuppressive signaling that collectively limit viral delivery, intratumoral spread, and therapeutic efficacy. The review provides an updated overview of clinical and preclinical studies of naturally occurring and engineered OVs, with a particular focus on the biological and translational challenges that restrict their effectiveness in HCC. In addition, it highlights the strategies developed to overcome delivery barriers, immune clearance, and tumor heterogeneity, which represent key obstacles to durable therapeutic responses. Strategies such as stromal targeting, hypoxia-adapted constructs, and the use of 3D organoid models as mimic platforms to evaluate delivery and therapeutic strategies are also discussed. Finally, this review aims to integrate recent advances in viral engineering, immune modulation, and organoid models, and critically evaluates how these approaches, together with emerging clinical trial data, can inform the rational design of next-generation oncolytic viruses.

**Supplementary Information:**

The online version contains supplementary material available at 10.1186/s12935-026-04331-1.

## Introduction

Oncolytic virus (OV) therapy is an emerging and rapidly developing area of research with significantly enhanced cancer cell targeting and minimal off-target effects. Unlike checkpoint inhibitors or adoptive cell therapies that solely modulate immune responses, OVs act as in situ vaccines by converting immunologically "cold" tumors into inflamed, immunogenic microenvironments amenable to immune-mediated clearance. The liver microenvironment, however, poses distinctive challenges for oncolytic virotherapy that extend beyond typical solid tumor barriers, making hepatocellular carcinoma both a significant unmet clinical need and a critical testing ground for next-generation OV strategies Hepatocellular carcinoma (HCC), the most common primary liver cancer and a leading cause of cancer mortality [1], presents a growing global burden driven by metabolic disorders [2] and chronic viral infections [3]. Despite advances in surgical approaches—including resection and transplantation and minimally invasive techniques [4], therapeutic options remain limited by organ shortages [5], disease complexity requiring multidisciplinary care [6, 7], and the frequent presentation of unresectable disease. While immune checkpoint inhibitors have shown promise [8], their efficacy is constrained by HCC’s immunosuppressive microenvironment. Oncolytic viruses offer a potentially transformative immunotherapeutic approach yet face substantial barriers unique to the hepatic context: Kupffer cell-mediated viral clearance, dense fibrotic stroma restricting penetration, and robust antiviral immunity that collectively limit therapeutic efficacy and necessitate innovative engineering solutions.

**Fig. 1:**
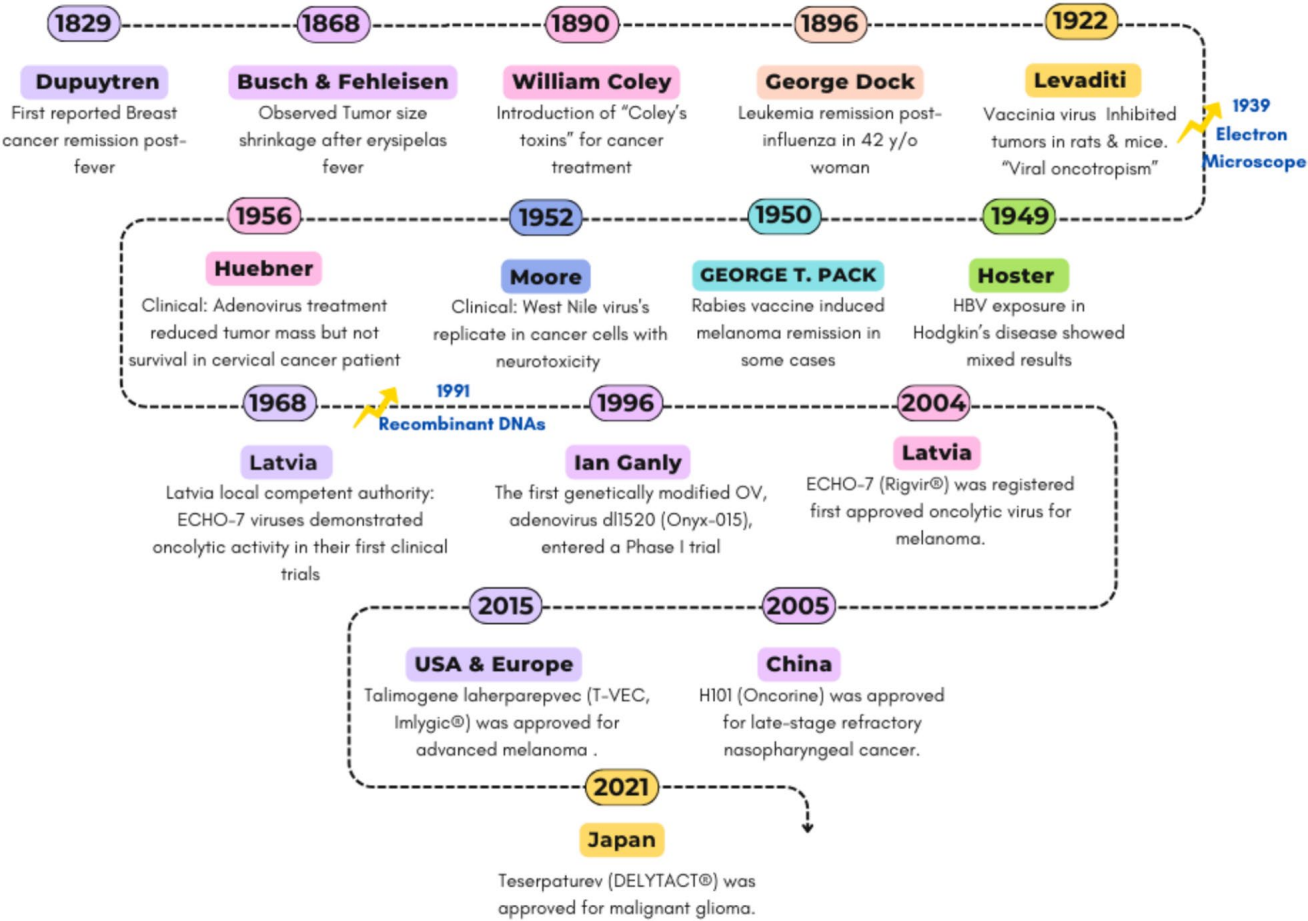
Historical timeline of oncolytic virotherapy. Major milestones from early observations (e.g., Coley’s toxins) to recent clinical approvals (e.g., T-VEC) are shown. OV: oncolytic virus

The origins of OV can be traced back to 1829, when Guillaume Dupuytren first observed a remission of breast cancer following fever. This observation was further reinforced in 1868 when two independent studies by Busch and Fehleisen, who reported tumor regression in patients with erysipelas infections [9, 10]. Significant progress was achieved in 1890 when William Coley introduced his revolutionary work on "Coley’s toxins," a mixture of killed bacteria that demonstrated significant success in treating inoperable tumors from 1890 through 1960 [11]. Coley’s toxins could be regarded as an early form of cancer immunotherapy and laid the groundwork for the use of biological agents, such as bacteria and viruses, to stimulate the anticancer immune response. Moving forward brings us to an important moment in cancer research in 1922 when Levaditi realized viral “Oncotropism”. He reported that the smallpox vaccine was able to inhibit various tumors in rats and mice. His work revealed that certain viruses have a natural specificity for cancer cells without affecting healthy ones, which could be attributed to the impaired pathways of the cancer cells [12]. Levaditi laid the groundwork for modern oncolytic virotherapy decades before the emergence of electron microscopy and the advancement of genetic engineering techniques. Throughout the mid-20th century, several clinical trials of OVs such as the West Nile virus and adenoviruses were performed but achieved limited success due to safety issues [13]. The field received another boost in the 1990s when recombinant DNA and genetic engineering methods were introduced, allowing viruses to be altered for improved safety and specificity. In 1996, the first genetically engineered OV to enter a phase I clinical trial was an adenovirus, Onyx-015 (dl1520) [14]. It was designed to target cancer cells with defective p53 pathways and achieved a considerable safety as well as antitumor activity. The entry of Onyx-015 into clinical trials opened the way for more sophisticated, engineered viral therapies. This was followed by several regulatory approvals marking OV therapy’s transition from an observational phenomenon to a sophisticated therapeutic approach [15, 16]. Figure 1 illustrates the key milestones in OV therapy, tracing its evolution from serendipitous clinical observations in the 19th century to the era of genetic engineering and regulatory approvals in the 21st century.

While OVs show immense promise as targeted cancer therapies due to their natural selectivity and immune-activating potential, their efficacy in dense, immunosuppressive solid tumors like HCC is often hampered by delivery barriers, rapid immune clearance, and tumor heterogeneity. Moreover, complementary approaches such as metal-based anticancer agents are being explored to synergize with OVs by overcoming chemoresistance and modulating the tumor immune landscape [17]. This review highlights that these hurdles can be overcome through genetic engineering to enhance specificity and immunomodulation, novel delivery strategies to navigate the tumor microenvironment (TME), and advanced testing platforms like 3D organoids, ultimately paving the way for next-generation OVs in hard-to-treat cancers.

### Review methodology and literature search strategy

In this article, a comprehensive review of clinical and preclinical literature on oncolytic virotherapy (OVT) was conducted. Electronic databases (PubMed/MEDLINE, Scopus, Web of Science) were searched for articles published between January 2000 and April 2025 using keywords and MeSH terms including “oncolytic virus,” “virotherapy,” “hepatocellular carcinoma,” “tumor microenvironment (TME),” “genetic engineering,” and “clinical trial.”

In parallel, a systematic search of the ClinicalTrials.gov database (U.S. National Library of Medicine) was performed using the terms “oncolytic virus” OR “virotherapy” in combination with “cancer.” All interventional trials registered up to April 2025 were screened without restrictions on phase, cancer type, or geography. Studies were included if they evaluated OVT for malignancy and reported results in the registry or linked publications. Observational studies and trials without results were excluded. To ensure transparent reporting, evidence is categorized as: Preclinical (in vitro, organoid, animal models) – interpreted as mechanistic insight. Clinical Phase I/II – indicating safety and preliminary efficacy. Clinical Phase III/approved – representing confirmatory efficacy and regulatory endorsement. Given the heterogeneity of OV platforms and trial designs, a qualitative narrative synthesis was employed. Data extraction focused on viral platform, cancer type, delivery route, and reported outcomes, summarized in Table 1 and Supplementary Table S2.

**Table 1:** Examples of genetically modified OVs

**Virus**	**Genome**	**Engineered virus name**	**Virus modification**	**Cancer type**	**Engineering approach**	**References**
NDV	ssRNA	NDV-MIP3α	Insertion of MIP-3α	colorectal carcinoma, melanoma	Dendritic cell recruitment to enhance antitumor immunity	[76]
		rNDV-cIFNγ	Insertion of cIFNγ	malignant oral melanoma	Induction of cellular immunity via interferon expression	[77]
		MEDI5395	Insertion of granulocyte-macrophage colony stimulating factor (GM-CSF)	Melanoma, colon carcinoma	Enhance monocyte activation	[78]
		rNDV-PTEN	Insertion of *PTEN*	Glioblastoma	Inhibits AKT/mTOR signaling to induce apoptosis	[79]
		MEDI9253	Insertion of *IL-12*	Colon cancer	Activates NK and cytotoxic T cells	[80]
		rZJ1-VS	V-deficient genotype VII recombinant NDV	Various cancers	Loses the ability to reduce phospho-STAT1 and induces higher expression of IFN-responsive genes.	[81]
Reovirus	dsRNA	rsT3D-L	Insertion of RGD peptide	Various cancers	Enhances cell tropism via integrin targeting	[82]
		Super Virus 5	Site-directed mutagenesis	Various cancers	Improves infectivity in cancer cells	[83]
		rS1-GMCSF	Insertion of murine /human GM-CSF	Pancreatic cancer	Stimulates dendritic and T cell activation	[66]
VSV	ssRNA	rVSV-UL141	Insertion of HCMV UL141gene	Multifocal HCC	Downregulates CD155 to escape NK-mediated clearance	[84]
		VSV-IFNβ-NIS	Insertion of IFNβ and NIS	Various cancers	Enhances selectivity and allows imaging	[85]
		rVSV-mIL12-mGMCSF	Fusion of IL-12 and GM-CSF	Glioblastoma	Activates innate/adaptive immunity via systemic delivery	[86]
		VSV-Δ51M-hIL-12	Δ51M deletion + IL-12 insertion	Various cancers	Reduces M protein toxicity; boosts immune infiltration	[87]
HSV	dsDNA	T-VEC	Deletion of RL and US12	Melanoma	Productive infection in malignant but not normal cells	[88]
		DELYTACT	Deletions in α34.5, α47, ICP6	Recurrent glioblastoma	Enhances NK and CD8+ T cell activation	[89]
		oHSV1-IL15B	Insertion of IL-15/IL-15Rα complex	Colon cancer	Stabilizes IL-15 improving the trans-presentation to NK cells and CD8+ T cells	[90]
		oHSV1-aPD-1	Insertion of anti-PD-1 antibody	Colon cancer	Checkpoint blockade with cytokine synergy	[90]
		G161	Insertion of IL-12, IL-15, PD-L1B	Breast cancer	Induces T, NK, and myeloid immune infiltration	[91]
Adenovirus	dsDNA	NG-641	Insertion of fibroblast activation protein-directed bi-specific T-cell activator antibody (FAP-TAc) and CXCL9/CXCL10/IFNα	Advanced or metastatic epithelial tumors	FAP-Tac targets immunosuppressive cancer-associated fibroblasts, while the CXCL9/CXCL10/IFNα activates the immune cells and recruits them.	[92]
		Ad5sPVR	Insertion of the soluble extracellular domain of poliovirus receptor (sPVR)	Various cancers	Overcomes the suppressive effects of checkpoints and insufficient costimulatory signals	[93]
		NSC-CRAd-S-pk7	Insertion of Survivin promoter (S) and fiber protein polylysine modification (pk7) loaded onto Neural stem cells (NSCs)	Glioma	The virus presents anti-neoplastic activity while bing loaded onto the NSCs, which preferentially migrate towards tumors.	[94]
		AdAPT-001	Deletion in Pea3 and E2F1 transcription factor sites. Insertion of a TGF-ß Trap.	Solid tumors	Selectivity enhancement.	[95]
		CG0070	Insertion of *GM-CSF*, E1A protein expression driven by human *E2F-1* promoter.	Bladder cancer	Preferential replication in Rb protein-defective cells, typical of bladder cancer, and GM-CSF produced activates the immune response.	[96]
Myxoma virus Myxoma virus (MYXV)	dsDNA	vPD-1/IL12	Insertion of soluble PD-1 inhibitor and IL12	triple negative breast cancer	The systemic function of vPD-1/IL12 is of high efficacy despite the low infection rates of the virus.	[97]
		vMyx-IL-15	Insertion of IL-15	Melanoma	Promotes infiltration of neutrophils which causes inflammation of the tumor bed.	[37]
		MYXVΔserp2	Deletion of *serp2*	Various types of cancer	Serp2 an anti-apoptotic and a virulence factor. The deletion enhances oncolytic effects and lessens the viral pathogenesis.	[98]
Senecavirus A	ssRNA	SVA-CH-01-2015	Insertion of p16^INK4A^	Various types of cancer	p16^INK4A^ will regulate the cell replication cycle and suppress tumor growth	[99]
		SVV-37	Insertion of CXCL9	Various types of cancer	CXCL9, known to help the recruit activated CD8+ and CD4+ cells	[100]
Parvovirus	ssDNA	H-1RGD	Insertion of the RGD-4C next to a nitro-carboxy group of Ala 441 of the VP2 sequence	Various Cancers	These residues prevented the entry of H-1PV into cells usually permissive for wild-type virus infection.	[47]
Vaccinia Virus	dsDNA	JX-594	Inactivation thymidine kinase (TK) insertion of *GM-CSF*	Various Cancers	Targets cancer cells through replication dependent cell lysis, and stimulation of antitumor immunity	[101]

## Naturally occurring OVs

Certain viruses can naturally infect cancer cells, which makes them candidates for oncolytic therapy. These viruses can bypass cellular signaling pathways and antiviral responses in cancer cells. Prominent examples include DNA viruses (Myxoma virus, Parvovirus H-1) and RNA viruses (Newcastle Disease Virus (NDV), Reovirus, Vesicular Stomatitis Virus (VSV)). The oncolytic properties of these viruses have demonstrated efficacy against malignancies, such as breast cancer, glioblastoma, melanoma, and neuroendocrine tumors. However, many factors, such as virus strain, tumor type, host immune condition, and administration mechanism, have a significant impact on treatment efficacy.

### Newcastle disease virus (NDV)

NDV is a non-segmented negative-sense single-stranded RNA virus that primarily infects avian species. NDV is non-pathogenic to humans and replicates only in the cytoplasm without integrating its genome into the host cell DNA [18]. NDV enters the targeted cells through receptor-mediated endocytosis [19]. The oncolytic potential of NDV was first observed by Cassel and Garrett in 1965 and was soon followed by extensive preclinical and clinical investigations [20]. NDV exploits interferon gamma (IFN-γ) deficiency in cancer cells to favor its genome replication and stimulate the secretion of numerous cytokines, including IFN-γ, IFN-α, TNF-α, and interleukin-1, produced by various immune cells such as natural killer (NK) cells, macrophages, and sensitized T cells that promote tumor destruction. Moreover, NDV infection has been reported to enhance MHC-I-mediated antigen presentation and the expression of several adhesion molecules such as ICAM-1 and LFA-3, contributing to the activation of T cells [21]. The immunosuppressive nature of the TME, especially in solid tumors, represents one of the prominent challenges that compromise the oncolytic potential of the NDV. These tumors possess physical barriers, including a dense extracellular matrix (ECM), which restricts viral infiltration and replication within the tumor mass and thereby reduces the therapeutic effect [22].

### Reovirus

Reovirus is a double-stranded RNA, non-enveloped virus with a segmented genome. It typically causes asymptomatic or mild enteric and respiratory illness, particularly in children. Its largely non-pathogenic nature in healthy humans enhances its safety profile. [23]. Reovirus targets cells through sialic acid residues and the junction adhesion molecules (JAM). It is selective toward cancer cells and depends on the activated RAS pathway, which downregulates PKR, a key cellular antiviral mechanism, allowing the virus to replicate preferentially in these cells. In addition, Reovirus can bind with high affinity to the N-terminus of EGFR, enhancing selectivity for cancer cells with activated EGFR and RAS pathways. Reovirus kills cancer cells by inducing apoptosis, which is triggered by IFN and NF-κB activation upon detection of dsRNA in the cytoplasm [24]. Reovirus showed efficacy against several types of cancers, including breast, colorectal, and pancreatic cancers. Although it is not approved by the FDA and EMA but showed promising results with ovarian and pancreatic cancer. One of the major obstacles in using Reovirus that limits its use in the clinical setup is the host immune response, tumor heterogeneity, and the lack of an optimal delivery system [25]. The host immune response rapidly clears the virus and limits its oncolytic activity. Moreover, there is a need for an effective delivery method that can ensure the virus reaches tumor cells in sufficient numbers and activity to exert its effect. Furthermore, the segmented genome of reovirus makes its genetic modification more challenging [25].

### Vesicular stomatitis virus (VSV)

VSV is a negative-sense, single-stranded, non-segmented RNA virus of the *Rhabdoviridae* family. It causes acute febrile vesicular diseases in livestock, mainly cattle and pigs and can infect humans, causing influenza-like symptoms. It selectively replicates in IFN-deficient human tumor cells, particularly those with mutant RAS and impaired PKR-mediated antiviral responses, while sparing normal cells. The AV2 strain is a strong oncolytic agent that can eradicate human ovarian carcinoma xenografts in mice [26]. In the mid-1960s, the oncolytic activity of VSV was shown to lyse lymphomatous tumors in mice [27]. VSV primarily enters cells via LDL receptors and alternatively via phosphatidylserine receptors, followed by uptake via endocytosis [28]. Neurotoxicity was reported in normal cells infected with VSV because of the virus’s ability to bind the LDL-R receptors. Other challenges, such as antiviral resistance and genetic instability, have also been reported, and the need for long-term safety follow-up has hindered clinical translation [29]. In addition, prolonged IFN activation has shown an increase in antiviral response and the adoption of an antiviral state by cancer cells, which compromises virotherapy.

### Adenovirus (AdV)

Adenovirus (AdV) is a non-enveloped, double-stranded DNA virus [30]. It was recognized as a prominent vector for gene therapy due to its high genetic stability and low pathogenicity. The oncolytic potential of adenovirus was first reported in the 1950s against HeLa cells, and since then, adenovirus has been extensively tested *in vitro* and *in vivo* models before reaching clinical trials [31]. The entry mechanism of the virus is achieved through receptor-mediated endocytosis. Viral entry is mediated by fiber knob binding to the coxsackie-adenovirus receptor followed by penton base interaction with integrins that induces clathrin-mediated endocytosis. The upregulated expression of the coxsackie-adenovirus receptor on cancer cells enhances the virus selectivity toward infecting cancer cells due to its crucial role in virus entry [32]. Upon entry, adenovirus is internalized into endosomes, then transported to the nucleus, where the viral genome is released and replicated, ultimately leading to cell lysis and spread to neighboring cells [31]. Adenoviral oncolysis has also been reported to target cancer stem cells, which are key players in cancer resistance. Adenoviruses have shown activity against several cancer types [33]. However, immune clearance, tumor non-specificity, and safety remain challenging to clinical application.

### Myxoma virus (MYXV)

Myxoma virus (MYXV) is a double-stranded DNA virus that naturally infects rabbits but not humans [34]. MYXV does not rely on a specific cell receptor-mediated entry but instead undergoes a multistep entry starting with cell attachment, membrane fusion, and delivery of the virion core into the cytoplasm [35, 36]. The MYXV replicates more efficiently in cells with activated AKT protein, due to phosphorylation at two specific sites on the AKT protein, threonine 308 (Thr308) and serine 473 (Ser473). When Thr308 is not phosphorylated, M-T5, a viral protein, compensates for that by inducing the phosphorylation of residue Ser473, enabling successful replication. Additionally, MYXV employs another viral protein, M062, to counteract SMAD9, an interferon-regulated protein that inhibits MYXV replication when active. Moreover, MYXV encodes M029, a dsRNA-binding protein that hinders the antiviral activity of the PKR protein. *p53* and *Rb* harbor mutations in cancer cells, subsequently leading to weak IFN and TNF response, allowing the virus to replicate, making the cells more permissive [37]. The MYXV was observed to have oncolytic activity against cancers such as melanoma [38], glioblastoma [39], gallbladder carcinoma [40], ovarian cancer [41], and small-cell lung cancer [37, 42]. However, the wild-type MYXV exhibits limited tropism for tumor sites, particularly the metastatic lesions.

### Senecavirus A (SVA)

Senecavirus A (SVA) or NTX-010 is a wild-type RNA virus member of the *Picornaviridae* family. SVA was accidentally isolated from the retinoblastoma cell line culture PER.C6. Pigs are established as the main hosts of SVA. SVA entry into the target cell is receptor-mediated and particularly dependent on tumor endothelial marker 8 (TEM8), which is overexpressed in neuroendocrine tumors [43]. Sialic acid-linked proteins and integrins may also contribute to viral attachment. SVA induces cancer cell death through intracellular replication and autophagy. It targets neuroendocrine-type tumors primarily, such as neuroblastoma, Wilms tumor, glioblastoma, and rhabdomyosarcoma [44, 45]. The virus has shown strong cytotoxicity against neuroblastoma, rhabdomyosarcoma, and Ewing sarcoma cells, with an IC50 as low as one virus particle per cell [44, 45].

### Parvovirus H-1 (H-1PV)

Parvovirus H-1 (H-1PV) is a small, non-enveloped, single-stranded DNA virus belonging to the Protoparvovirus genus. Rodents are the natural hosts of H-1PV [46]. Studies on the oncolytic activity of H-1PV started in the early 1960s and continued for decades. It has selectivity toward cancer cells with impaired antiviral defense mechanisms. H-1PV employs sialic acid residues on the cell surface for cell attachment. Laminin, an important component of the ECM, was also identified as a cofactor helping facilitate viral binding and entry through clathrin-mediated endocytosis [47, 48]. H-1PV replicates preferably in cancer cells that overexpress E2 transcription factors, CREB, ATFs, and cyclin A, which are all essential for viral DNA replication and gene expression. In addition, the non-structural protein NS1 of the virus, which is important for viral DNA replication, is also regulated by post-translational modifications such as phosphorylation and acetylation in the host cell, which consequently influence its activity in tumor cells [49]. Besides its direct oncolysis, H-1PV induces antitumor immune responses, converting the TME into an immunogenic state. The first clinical evidence of its oncolytic activity was established in the ParvOryx01 (wild-type H-1PV clinical grade) phase I/IIa trial in patients with recurrent glioblastoma. It was also shown that the ParvOryx01 has the potential to cross the blood-brain barrier and extensively distribute within tumors to induce immune responses involving microglia/macrophage activation, cytotoxic T-cell activation, and infiltration [50]. Despite its therapeutic potential, the dense extracellular matrix (ECM) can act as a physical barrier to viral spread, limiting efficient tumor penetration. Additionally, while the virus selectively targets cancer cells, enhancing its specificity and efficacy remains an area for improvement.

### Vaccinia virus (VACV)

The vaccinia virus (VACV) is an enveloped double-stranded DNA virus that belongs to the Orthopoxvirus genus of the *Poxviridae* family. Initially, it was used as a smallpox vaccine [51]. Its safety in humans is due to its exclusive cytoplasm replication, which lacks integration into the host genome [52]. VACV entry is versatile: while it does not require a single, specific receptor, its entry is mediated by mechanisms like macropinocytosis and facilitated by various attachment factors. This flexibility allows it to infect a wide variety of cell types, including cancer cells. It is important to note that specific entry pathways and receptor usage can vary depending on the VACV strain and cellular context [53]. The tumor tropism of VACV is attributed to the downregulation of tumor suppressor genes and antiviral pathways such as IFN-I, which under normal conditions restricts viral replication in normal cells [54]. VACV also induces strong immunogenic responses by activating host immune molecules, further increasing its antitumor activity. VACV shows oncolytic potential against pancreatic [55] and lung cancers [56]. The oncolytic activity of the virus is achieved via direct oncolysis of tumor cells, vascular disruption (which includes lysis of tumor-associated endothelial cells and thrombosis caused by neutrophil accumulation), and induction of antitumor immunity [57]. Despite its potential, the immune system can mount antiviral responses that can limit viral persistence and activity. Upon cytoplasmic entry, the cyclic GMP-AMP synthase/stimulator of interferon genes (cGAS/STING) pathway is activated, triggering antiviral cytokine production such as IFN-I that inhibits VACV replication and causes viral degradation [58].

### Maraba virus

Maraba virus is a negative-sense, single-stranded RNA virus belonging to the Rhabdoviridae family, closely related to vesicular stomatitis virus (VSV). Its oncolytic activity stems from its preferential replication in cancer cells with defective type I interferon (IFN) signaling pathways, a common feature in many malignancies. Maraba virus enters cells via the low-density lipoprotein receptor (LDL-R) and induces rapid cytolysis. It has demonstrated potent oncolysis in preclinical models of various solid tumors, including sarcoma, melanoma, and non-small cell lung cancer. A key feature of Maraba virus is its ability to induce robust, antigen-specific CD8+ T-cell responses against tumor-associated antigens, effectively acting as an in situ cancer vaccine. Clinical evaluation of an attenuated, recombinant Maraba virus strain (MG1) is underway, with early-phase trials exploring its safety and immunogenicity [59]. A primary challenge for systemic administration is its sensitivity to pre-existing anti-viral antibodies and rapid clearance by the reticuloendothelial system.

### Oncolytic herpes simplex virus (oHSV)

Oncolytic herpes simplex virus type 1 (oHSV) is a genetically engineered DNA virus and one of the most clinically advanced OV platforms. Wild-type HSV-1 is a neurotropic human pathogen, but through targeted deletions of virulence genes (e.g., ICP34.5 and ICP47), its replication can be restricted to cancer cells while enhancing anti-tumor immune responses. oHSV strains, such as talimogene laherparepvec (T-VEC), are further armed with immunostimulatory transgenes like granulocyte-macrophage colony-stimulating factor (GM-CSF). The virus exploits upregulated cellular receptors (e.g., nectin-1) on cancer cells for entry and induces immunogenic cell death. T-VEC is approved for the local treatment of unresectable melanoma and has shown promise in other solid tumors. Its ability to establish latent infections in neurons allows for persistent antigen presentation, but this also necessitates careful engineering to ensure safety and prevent reactivation. Clinical success has been particularly noted in immunologically responsive tumors, though efficacy in highly immunosuppressive or fibrotic microenvironments remains a challenge [60].

Although these natural OVs demonstrate potent oncolytic activity, their efficacy is often constrained by immune evasion issues and poor tumor penetration, challenges that genetic engineering strategies aim to resolve, as discussed next.

## Genetic engineering approaches to enhance the natural OV efficiency

**Fig. 2:**
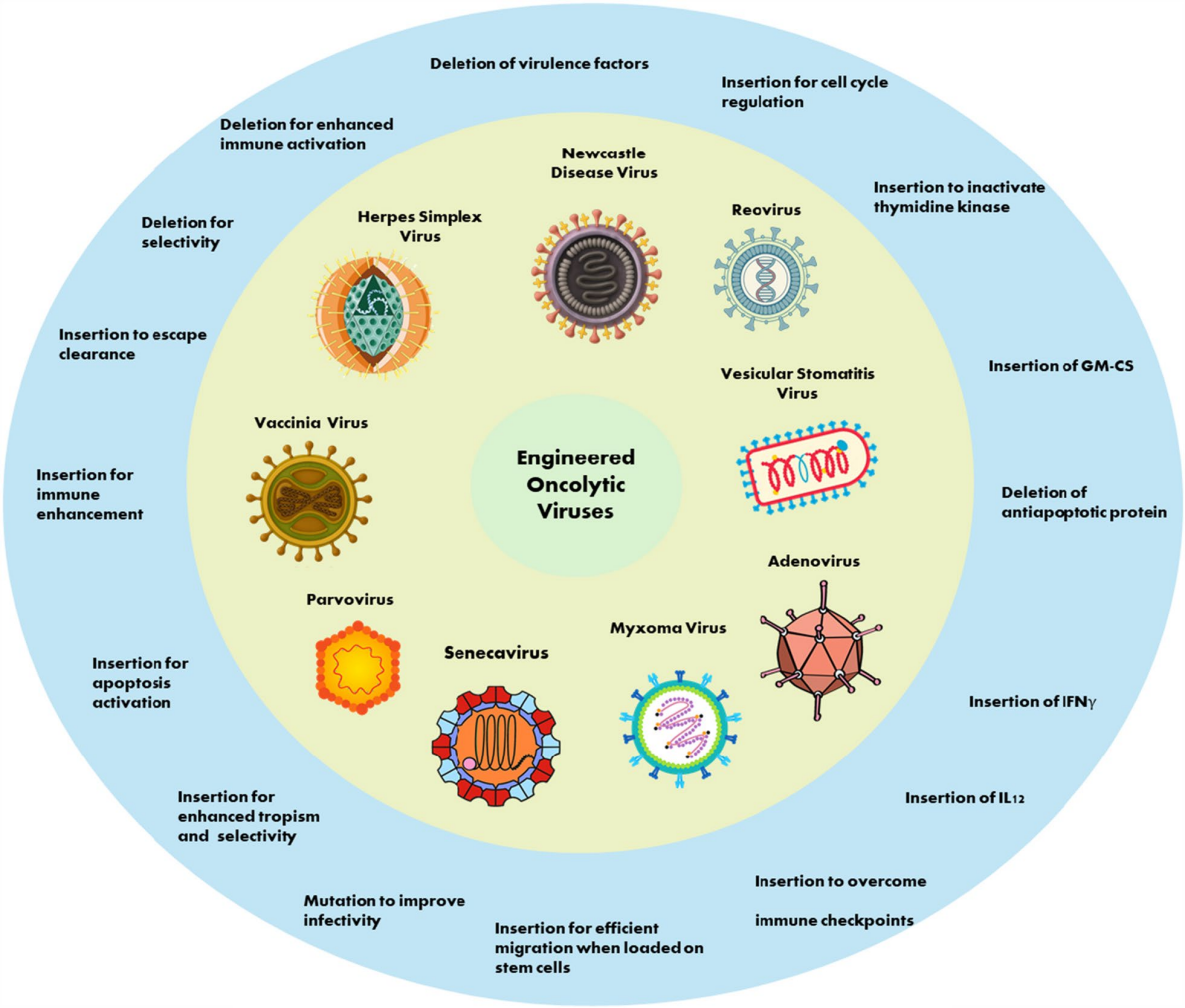
Genetic engineering approaches to enhance oncolytic virus efficacy. Schematic representation of common modifications across OV platforms: gene insertions (e.g., immunostimulatory cytokines like GM-CSF, IL-12, IFNγ) and deletions (e.g., viral antiapoptotic or virulence genes) to improve tumor selectivity, immune activation, and delivery. Abbreviations: GM-CSF, granulocyte-macrophage colony-stimulating factor; IL, interleukin; IFN, interferon. The figures of viruses were generated using open AI image generation and were verified for scientific accuracy, the adenovirus figure was obtained from NIH/NIAID Bioart

Various engineering strategies have been adopted to overcome the limitations of natural oncolytic virotherapy outlined in Sect. "Naturally occurring OVs", such as rapid immune clearance, suboptimal tumor specificity, antiviral responses (e.g., via IFN or PKR pathways), and poor penetration in immunosuppressive tumor microenvironments (TMEs), while amplifying antitumor efficacy (Fig. 2). Figure 2 provides a schematic overview of the principal genetic engineering approaches, including gene insertions (e.g., cytokines, receptors) and deletions (e.g., of viral virulence factors), applied across diverse OV platforms to enhance tumor selectivity, immune activation, and replication.

Below, we synthesize the most promising strategies for key viruses, emphasizing why approaches like cytokine arming and promoter-driven targeting are effective and how they directly address natural OV constraints. Table 1 summarizes representative examples, highlighting the shift from broad-spectrum natural OVs to tumor-tailored constructs, with detailed constructs and outcomes in Supplementary Table S1.

For RNA-based OVs like NDV, which naturally exploits IFN-deficient cancer cells but faces rapid clearance and limited immune stimulation (Sect. "Naturally occurring OVs"), cytokine arming stands out as a highly promising strategy. Recombinant NDV has been engineered to produce immunomodulatory substances like interferons, interleukin-2 (IL-2), granulocyte-macrophage colony-stimulating factor (GM-CSF), tumor necrosis factor-alpha, immunoglobulin G, and complement regulatory proteins to enhance immune responses against tumors [61]. In addition, genetic modifications to introduce a hypercleavage F protein were reported to enhance selectivity for tumors as well as syncytium formation, thereby increasing cytotoxicity toward tumors [62]. In another approach, NDV has been genetically modified to make it sensitive to type I interferons to increase its selectivity for cancer cells [63]. This is effective because it leverages NDV’s cytoplasmic replication for local immunostimulatory payload delivery, minimizing systemic toxicity while addressing Sect. "Naturally occurring OVs"‘s immune evasion issues—preclinical studies show superior tumor regression in HCC models compared to unmodified NDV, especially when combined with metabolic modulators like dichloroacetate to overcome TME suppression [64].

Genetic modifications like hypercleavage F protein introduction further boost cytotoxicity by promoting syncytium formation, improving selectivity and overcoming delivery barriers in dense tumors [51].

Reovirus (Reo), limited by its segmented genome structure and JAM-dependent entry benefits most from reassortment and cytokine arming. A significant breakthrough involved developing JAM-A independent entry strains that overcome JAM-A entrance dependency, sensitizing the virus to JAM-negative cells and expanding the range of targetable cancer cells [65]. A recombinant virus expressing GM-CSF has been constructed to modulate anticancer immune responses. Co-infection with several REO virus strains was performed to generate reassorted viruses with improved infectivity against aggressive cancer cells, including triple-negative cancers [66]. Moreover, induced mutations in viral protein σ1 protect the virus from protease activities and increase viral lifetime, enhancing therapeutic efficacy [67]. Developing JAM-A-independent strains expands targeting to receptor-negative cancers, addressing specificity constraints and enhancing infectivity in aggressive tumors like triple-negative breast cancer [55, 56]. GM-CSF expression modulates immune responses, converting "cold" TMEs to "hot" ones by recruiting antigen-presenting cells (APCs), which counters antiviral clearance and boosts efficacy, mutations in σ1 protein prolong viral lifetime against proteases, directly mitigating Sect. "Naturally occurring OVs"‘s host response limitations [57].

By rerouting viral attachment to cancerous cells, the genetic modification that allows the expression of single-chain antibodies against tumor-associated antigens such as Her2/neu, PSMA, EGFR, GD2, CEA, and MUC1 improves selective cytotoxicity and decreases off-target effects in VSV. Complementing this targeting approach, the exchange of VSV glycoproteins with those from other viruses, such as Sindbis virus, measles virus, CHIKV, and LCMV, alters viral tropism, allowing tumors to be selectively infected and preexisitng immune responses to be evaded. Moreover, gene insertion techniques have been used to incorporate immunostimulatory genes like IL-12, GM-CSF, IFNβ, and NIS (sodium iodide symporter) that augment anti-tumor immune responses and allow noninvasive tracking of tumors treated with the virus [68]. Furthermore, attenuation of the M gene, like the ΔM51 or M51R gene, lessens the viral inhibition of interferon signaling, which increases targeted replication in cancerous cells with compromised immunity, while lowering toxicity. Since single-cycle VSV pseudoviruses replicate once, they offer an even safer platform, lowering toxicity while maintaining immune-mediated antitumor activity. Vesicular stomatitis virus (VSV), hindered by neurotoxicity and antiviral IFN inhibition (Sect. "Naturally occurring OVs"), excels with glycoprotein exchange and M gene attenuation as promising approaches. Swapping glycoproteins (e.g., with LCMV or measles) reroutes attachment to tumor antigens like HER2 or EGFR, improving specificity and evading antibodies, which complements attenuation strategies like ΔM51 to reduce IFN suppression and lower toxicity while maintaining immune-mediated antitumor activity [58]. These are effective for their balance of safety and potency, enabling noninvasive tracking via NIS insertion and addressing off-target effects in preclinical models.

DNA-based OVs, such as adenovirus, present distinct genetic engineering opportunities and challenges in contrast to the RNA viruses. The construction of ONYX-015 with a deleted *E1B* 55-kDa-associated protein, marked the beginning of adenovirus genetic engineering. This alteration makes the virus benign in healthy cells with intact p53 while infect cancerous cells with a non-functional p53 [69]. For adenovirus (AdV), promoter-driven targeting (e.g., AFP or hTERT promoters) restricts replication to p53/Rb-defective HCC cells, enhancing specificity and reducing hepatotoxicity, this is particularly effective as it exploits cancer-specific defects, overcoming Sect. "Naturally occurring OVs"‘s off-target concerns, with ONYX-015’s E1B deletion marking a foundational success in clinical safety [59]. Large DNA viruses such as MYXV offer a unique platform for genetic engineering due to their large genomic capacity. Genetic modification of MYXV primarily focused on enhancing OV effectiveness via the inactivation of genes responsible for particular viral death modifiers [37]. Furthermore, the engineering of MYXV to express soluble ectodomain form of PD-1 (sPD-1) provides a more effective and less harmful tumor-specific cytotoxic T cell response compared to standard cancer treatments with anti-PD-1 antibodies, thus highlighting the potential of MYXV-based delivery of other checkpoint inhibitors like CTLA-4, LAG-3, and TIM-3 [70]. Myxoma virus (MYXV) benefits from checkpoint inhibitor arming, such as soluble PD-1 expression, which boosts tumor-specific T-cell responses more effectively than systemic antibodies, addressing TME immunosuppression while leveraging AKT-dependent replication for specificity in melanoma and ovarian models [28, 60].

Similarly, engineered Seneca Valley Virus (SVA) has emerged as a prominent candidate for oncolytic virotherapy. SVA enhancement involves genetic modifications that allow it to function as a vector for therapeutic molecules, including cytokines like GM-CSF and IL-24, as well as antibodies against tumors, thus promoting SVA as an effective delivery system for oncological therapies [71]. Utilizing the virus’s evolutionary characteristics, SVA is modified to express tumor-associated antigens, which advances the development of more targeted and potent cancer therapies. Engineered Senecavirus A (SVA) prioritizes cytokine arming (e.g., GM-CSF, IL-24), functioning as a vector for tumor-associated antigens to advance targeted therapies—this counters Sect. "Naturally occurring OVs"‘s limited immune activation, promoting SVA as a safe delivery system for neuroendocrine cancers [61].

Another example is H-1PV (Parvovirus H-1), which can be modified to optimize viral entry, replication, and immune modulation. A modification must delicately balance the trade-off between maintaining safety profiles and achieving maximum efficacy. The discovery of host factors that are required for virus entry and replication represents a significant advancement in the clinical translation of H-1PV-based oncolytic therapy. For parvovirus H-1 (H-1PV), optimizations in entry/replication via host factor discoveries balance safety and efficacy, addressing antiviral barriers and enabling clinical translation in glioblastoma [42].

In addition to traditional recombination-based methods, CRISPR/Cas systems have emerged as a versatile and precise tool for the genetic modification of OVs [72]. This technology facilitates targeted knock-in of therapeutic transgenes (e.g., cytokines, bispecific T-cell engagers) or knock-out of viral virulence and host antiviral genes, thereby enhancing tumor selectivity, safety, and immunostimulatory capacity. For instance, CRISPR/Cas9 has been employed to engineer oncolytic adenoviruses with armored cytokine expression, demonstrating improved antitumor efficacy and a favorable safety profile. For example, Zhang team reported the use of CRISPR/Cas9 to engineer an oncolytic HSV-1 by replacing the ICP34.5 locus with the immunomodulatory genes IL-12 and CXCL11 and deleting ICP47, resulting in enhanced antitumor efficacy in colorectal cancer models [73]. In another study, genome-wide CRISPR screening can identify host restriction factors such as PARP1, whose inhibition enhances oncolytic HSV-1 replication and antitumor efficacy, enabling synergistic combination strategies with PARP inhibitors and immune checkpoint blockade in GBM and TNBC [74]. Similarly, CRISPR interference (CRISPRi) can be used to downregulate host factors that restrict viral replication, selectively potentiating OV activity in cancer cells wich remains emerging area of research [75]. These precision editing approaches expand the genetic engineering toolkit, enabling the creation of next-generation OVs with tailored functionalities to overcome specific tumor microenvironment barriers.

These engineering innovations significantly boost OV selectivity and potency; however, even engineered OVs must contend with host immune clearance mechanisms, which we explore in the following section to underscore the need for complementary immunomodulatory approaches.

## Host immune mechanisms limiting oncolytic virus persistence and activity

### The innate-immune dilemma in OV therapy: from viral clearance to antitumor immunity

The therapeutic success of oncolytic virotherapy hinges on a critical balance within the host immune system. The same innate immune pathways that rapidly detect and attempt to eliminate the virus are also responsible for initiating the desired, sustained antitumor immune response. Understanding this dual role, where the host’s defenses act as both the primary barrier and an essential partner, is key to designing effective OVs**.**

Upon administration of oncolytic viruses (OVs), the host innate immune system initiates an immediate alarm response through pattern recognition mechanisms mediated by pattern recognition receptors (PRRs), which survey infected cells for conserved viral components known as pathogen-associated molecular patterns (PAMPs) [102, 103]. Cytosolic RNA sensors, including retinoic acid–inducible gene I (RIG-I) and melanoma differentiation-associated protein 5 (MDA5), detect distinct forms of viral RNA within the cytoplasm that are characteristic of RNA virus replication, such as Newcastle disease virus (NDV), vesicular stomatitis virus (VSV), and reovirus infections [104, 105]. In parallel, cytosolic viral DNA is sensed by the cyclic GMP-AMP synthase–stimulator of interferon genes (cGAS–STING) pathway, which is typically activated during DNA virus infections, including those caused by adenovirus and vaccinia virus [54]. Viral nucleic acids are also detected within endosomal compartments by Toll-like receptors (TLRs), notably TLR3, which recognizes double-stranded RNA, TLR7/8, which sense single-stranded RNA, and TLR9, which detects CpG-rich DNA motifs [102, 103]. Additionally, protein kinase R (PKR) functions as both a sensor and an effector molecule by directly binding cytoplasmic double-stranded RNA, thereby contributing to the antiviral response following OV infection [24].

#### Signaling cascades and interferon induction following PRR engagement (Amplifying the alert)

Engagement of PRRs by oncolytic viruses activates potent and convergent signaling pathways that amplify the innate immune response [104]. Viral RNA sensing by RIG-I or MDA5 and viral DNA recognition through the cGAS–STING axis lead to activation of the adaptor proteins MAVS and STING, respectively, which converge on the downstream kinases TBK1 and IKKε [58]. These kinases phosphorylate the transcription factors IRF3 and IRF7, promoting their nuclear translocation, while parallel signaling pathways activate NF-κB [58] [102, 103, 106]. The coordinated activation of these transcriptional programs results in robust induction of type I interferons (IFN-α/β) together with pro-inflammatory cytokines, including TNF-α and IL-6, thereby amplifying the antiviral alert response [104].

#### Induction of an antiviral state through interferon signaling (Shutting down the factory)

Secreted IFN-α/β act in both autocrine and paracrine manners by binding to the IFN-α/β receptor (IFNAR), which activates the JAK–STAT signaling pathway [102, 103, 106]. This signaling cascade induces the expression of hundreds of interferon-stimulated genes (ISGs), establishing a potent antiviral state in infected cells as well as in surrounding uninfected cells [104] [102, 103, 106]. Among the key ISG effectors is protein kinase R (PKR), which is activated by double-stranded RNA and phosphorylates the eukaryotic translation initiation factor eIF2α. This phosphorylation event causes a global inhibition of cellular protein synthesis, effectively halting viral replication while simultaneously imposing substantial stress on the host cell.

#### The therapeutic paradox of viral clearance and immune activation (Clearance vs. Activation)

These innate immune processes underscore a central paradox in oncolytic virus therapy [102–104, 106]. On one hand, rapid and systemic induction of type I interferons and ISGs such as PKR promotes efficient antiviral clearance, leading to neutralization of viral particles, elimination of infected cells, and establishment of an antiviral microenvironment that can markedly restrict OV replication, intratumoral spread, and overall therapeutic efficacy, particularly following systemic administration [107, 108]. On the other hand, the same inflammatory response is essential for therapeutic benefit [104] [58]. The cytokine and chemokine milieu recruits innate immune cells, including natural killer cells, dendritic cells, and macrophages, while virally induced immunogenic cell death of tumor cells results in the release of tumor-associated antigens and damage-associated molecular patterns [109, 110]. This process converts an immunologically “cold” and suppressive tumor microenvironment into an inflamed, immunogenic state that supports antigen presentation and the priming of tumor-specific cytotoxic T lymphocyte responses, thereby contributing to durable antitumor immunity [104].

#### Exploiting the balance for selectivity

OVs are engineered to exploit the common defects in these antiviral pathways found in cancer cells [102, 103]. Tumors often have deficient IFN signaling, inactivated PKR (e.g., due to oncogenic RAS signaling), or impaired apoptosis—all of which render them permissive for viral replication while normal cells activate the full antiviral arsenal [24] [102, 103]. Thus, the genetic design of OVs aims to attenuate these antiviral pathways (e.g., deleting viral IFN antagonists) for safety in normal tissue, while preserving or enhancing their ability to trigger ICD and inflammatory signaling within the immunodeficient tumor [102, 103].

In summary, the innate immune response to OVs is a double-edged sword. The initial goal of the host is to clear the infection via the IFN/PKR-mediated antiviral state. The goal of therapy is to harness and redirect this inflammatory response to achieve immunogenic tumor cell killing and establish systemic antitumor immunity [102, 104] . The next section details how this interplay dictates the pharmacokinetic and pharmacodynamic challenges of OV therapy.

### Adaptive immune responses and clearance mechanisms

Upon entry of OVs into the body, virus particles become coated with neutralizing antibodies and removed [111]***.*** Infiltrating innate immune cells and antigen-specific T cells can also destroy infected tumor cells, thus terminating OV infection before full therapeutic benefit can be achieved [112]. The presentation of viral or tumor-associated antigens (TAA) to cells of the adaptive immune system induces antigen-specific cellular and humoral immune responses. The primary antitumor effector cells CD8+ CTLs are critical mediators of OV-induced antitumor immunity. After recognizing specific antigens on the membranes of cancer and infected cells that are displayed on MHC class I molecules, they discharge perforin and granzymes to kill the cells. APCs stimulate CD4+ T-helper cells, which play a significant role in antitumor immunity by secreting pro-inflammatory cytokines and inducing CTL formation. Following exposure to the virus particle, humoral immune responses are initiated, and B cells, once activated, yield immunoglobulins. Neutralizing or opsonizing antibodies inhibit viral function and promote elimination [113, 114].

Upon administration of OVs, viral elements known as pathogen-associated molecular patterns (PAMPs), such as proteins, DNAs, RNAs, and viral capsids, are exposed to the host immune system, causing immunogenic cell death (ICD) [102]***.*** Inflammatory pathways in liver cancer, particularly those mediated by cytokines and chemokines, critically influence the immunogenic response to OVs and represent actionable targets for combination therapy [115]. ICD provides the basis for OVs to elicit antitumor immunity, which includes not only apoptosis but also necroptosis, necrosis, autophagic cell death, and pyroptosis of cancer cells [110]. Furthermore, the production of TAAs and tumor-associated neoantigens (TANs) contributes to ICD [109].

PAMPs/DAMPs also increase the production of cytokines and chemokines such as IFNs, TNF-α, IL-6, IL-1, CCL2, CCL3, CCL5, and CXCL10 [102]***.*** Chemokines and cytokines play a role in attracting neutrophils and macrophages to infection sites, as well as boosting the activity of NK cells and DCs, which further increases the innate response and transforms immunologically "cold" tumors into "hot" tumors [103]***.*** OVs also stimulate adaptive immunity against tumor cells, primarily via the tumor-specific T-cell response [104]. and can remodel the tumor microenvironment to enhance T-cell infiltration and function [116]. Tumor cells infected with the measles virus vaccine or treated with Reovirus virotherapy can overcome evasion of antigen presentation and enhance cross-presentation of tumor antigens by human plasmacytoid dendritic cells [117]***.*** IFNs activate MHC class I and II molecules, as well as costimulatory molecules on the surface of DCs, such as CD40, CD80, and CD86, after OVs infect tumor cells [118]. Many OVs, including HSV-1, OVV, VSV, MeV, and OAd, can induce antigen-specific T-cell immunity against malignancy [119]. However, neutralizing antiviral antibodies, either preexisitng or induced by treatment, may inhibit OVs from replicating within and lysing tumor cells [107, 120]. The balance between therapeutic immune activation and detrimental preexisting antiviral immunity remains a critical determinant of OV efficacy [116].

Additionally, antiviral mechanisms of resistance like complement activation, antiviral cytokines, and macrophage responses can rapidly eradicate OVs [106, 121]. Although the full effect of antiviral immunity remains unknown, OVs may have serious challenges because of these immune responses. Intratumoral administration of OVs may help overcome this limitation by inducing both local and abscopal antitumor effects. Surprisingly, although preexisitng immunity to NDV limits its replication within tumors, it does not impair tumor clearance, abscopal antitumor immune responses, or survival. These outcomes are even enhanced in NDV-immunized mice receiving repeated treatment doses [120].

The host immune system efficiently clears viruses, which can reduce the effectiveness of viral therapy. In such cases, the immune response eliminates the virus before it can reach and act on the target tissue, particularly affecting systemically administered viral therapies.

Furthermore, hyperactivation of the immune system in response to virus therapy might cause systemic inflammation or other negative immune-related effects [91, 92]. Induction of a systemic antitumor immune response and selective replication within tumor cells, resulting in the cell death within hours, have been suggested to be the two main mechanisms of the anticancer effect of OVs. Intracellular Toll-like receptors (TLRs), which detect pathogen-associated molecular patterns (PAMPs) such as viral capsids, DNA, RNA, and proteins, are activated when cells are infected by an OV. This activation triggers the JAK-STAT and NF-κB pathways, resulting in the production and secretion of type I interferons [93].Toll-like receptors (TLRs) and type I interferons both activate the interferon-induced, double-stranded RNA-dependent protein kinase (PKR), which is essential for regulating cell division and the stimulation of natural antiviral defense. Cell division and viral replication are blocked when PKR activation suppresses cellular protein synthesis [93]. The next-generation OVs transcending current limitations are being made possible by innovation in synthetic biology technology and immuno-oncology [93].

Understanding these immune barriers is crucial for optimizing OV therapy in complex TMEs, particularly in HCC where stromal and hypoxic elements further complicate delivery, topics we address to illustrate targeted strategies for overcoming tumor-specific hurdles.

## The impact of the TME on OV delivery in HCC

HCC is one of the most common primary liver cancers and the leading cause of cancer-associated mortality, making it a significant global health burden [1]. Emerging immunomodulatory strategies beyond checkpoint inhibition are reshaping how the immunosuppressive TME can be targeted to potentiate OV efficacy in HCC [^2^. Activated Hepatic stellate cells (HSCs), under the influence of inflammatory signals, result in the formation of scar tissue, which in turn creates a favorable environment for the onset of HCC. The chronically inflamed environment influences the accumulation of somatic alterations and impaired cell regeneration, along with blockage of sinusoidal cavities due to the collagenized tissue; all these factors contribute to the progression from cirrhosis to HCC [122]. This process is usually accompanied by a dramatic change in the tissue architecture and tissue-resident immune cell compartments [123]. The extracellular matrix (ECM) is a key player in hepatocellular carcinoma (HCC) TME dynamics. The ECM is the macromolecular complex network providing structure and regulating cellular processes in tissues. It is made up of hundreds of proteoglycan macromolecules, glycoproteins, collagen fibers, ECM regulators such as LOX, and metalloproteases (MMPs) [124].

The TME of HCC comprises both immune and nonimmune compartments in addition to the ECM. ECM remodeling is a continuous and dynamic process within the hepatic tissue, starting from the inflammation phase through fibrosis and reaching cirrhosis and HCC. This remodeling is accompanied by altered mechanics and metabolic changes in the cells and in turn affects the immune cells’ response and functionality [124]. Starting with the early fibrosis, HSCs are hyperactivated and transdifferentiate into myofibroblasts that release high levels of collagen I and collagen III proteins [125]. However, in later stages, the elastin and collagen fibers start to intertwine to form a strong and non-repairable ECM, which cannot be easily degraded by metalloproteases. This increased deposition of closely cross-linked fibrinogen, which is highly colocalized with collagen, forms a dense fibrous network leading to the development of cirrhotic tissue.

**Fig. 3:**
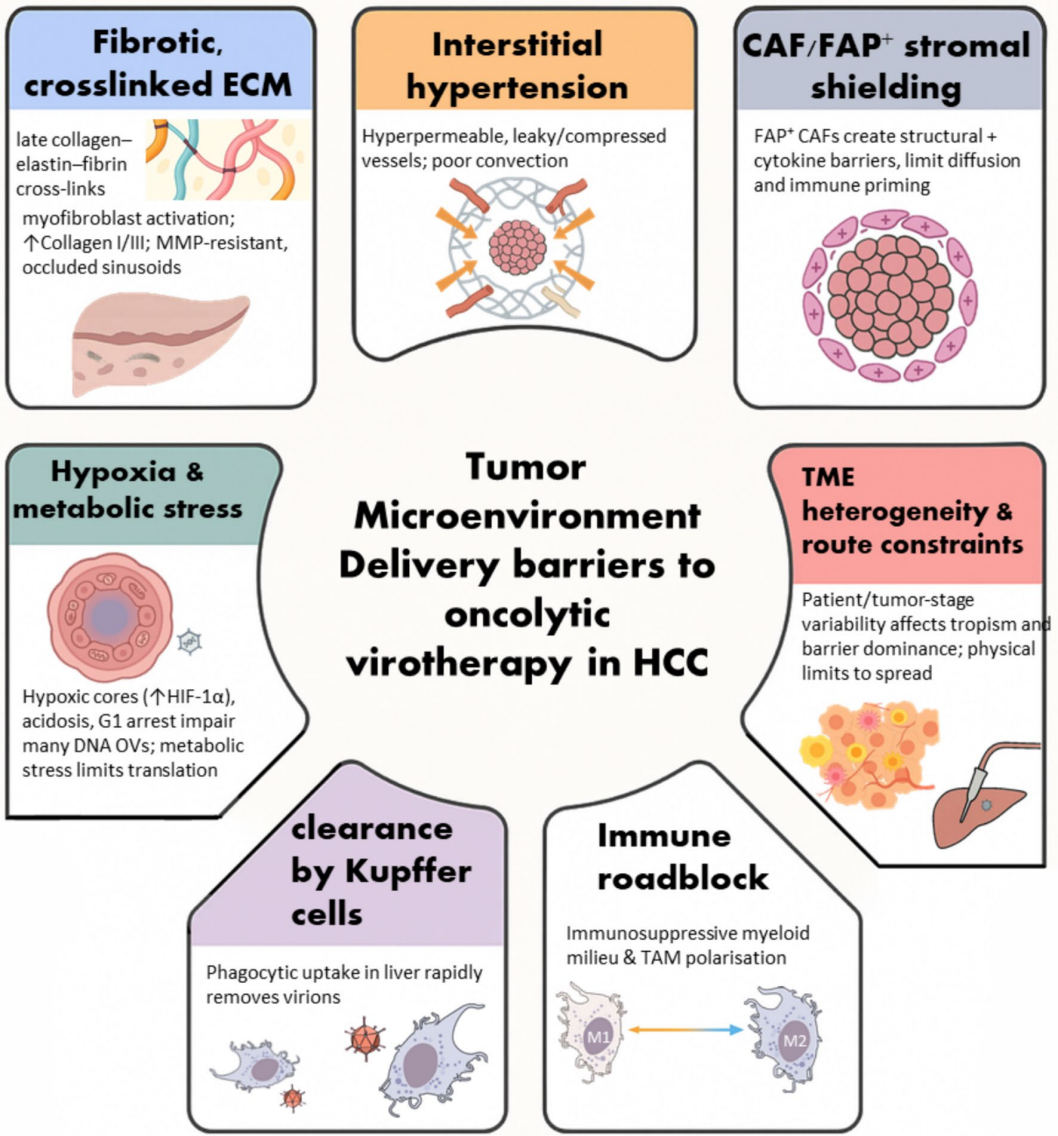
Major barriers to OV delivery in hepatocellular carcinoma (HCC). The tumor microenvironment (TME) presents physical (e.g., dense extracellular matrix (ECM), interstitial pressure), immunological (e.g., Kupffer cell sequestration, immunosuppressive macrophages), and stromal (e.g., cancer-associated fibroblasts (CAFs)) barriers that limit viral penetration, replication, and efficacy. Hypoxia and metabolic stress further complicate therapeutic delivery. Abbreviations: ECM, extracellular matrix; CAFs, cancer-associated fibroblasts; TME, tumor microenvironment. All the drawings included are conceptualized using AI-generated illustrations (created by the authors) and rigorously verified for scientific accuracy by the authors, except the clearance by Kupffer cells and immune roadblock part images were obtained from NIH/NIAID Bioart

The penetration of this TME is one of the major challenges facing OV therapy due to the adverse conditions that the virus faces (Fig. 3). The dense extracellular matrix (ECM) in fibrotic HCC creates physical barriers, limiting both the efficient delivery and subsequent spread of the virus within the tumor. The fibrotic ECM, accompanied by vascular hyperpermeability, leads to the development of interstitial hypertension, further limiting OV delivery in HCC [126]. Research efforts are being made to develop armed OVs that can overcome the hurdles of the ECM. For example, genetically engineered OVs target FAP, which is expressed predominantly by stromal and cancer-associated fibroblasts. Yu et al [127] genetically engineered a VACV (mFAP-TEA-VV) with anti-FAP bispecific T cell engager expression, enabling T cells to target tumor cells as well as FAP-expressing stromal cells. This virus caused more inhibition of tumor growth compared to control viruses targeting tumor cells alone in the B16 melanoma mouse model. However, as this tumor model was established within a short timescale, it is unlikely to be representative of tumor heterogeneity and complexity in human disease. In addition, the expression of FAP on other stromal cells makes off-target effects likely [127]. Building on this concept, Freedman et al [128] engineered EnAd-FAP-BiTE, a genetically engineered enadenotucirev adenovirus for the secretion of an anti-FAP BiTE following replication in tumor cells. The virus was successful in activating T cells in malignant ascites and prostate cancer biopsies, depleting FAP+ cells, and eliminating the immunosuppressive cytokine TGF-β. Additionally, transcriptomics validated the upregulation of pro-inflammatory cytokines, enhanced T cell functionality, as well as enhanced chemokine levels for T cell recruitment. Despite these encouraging findings, the exclusive targeting of CAFs remains unclear. In another study, an oncolytic adenovirus (ICO15K-FBiTE) was genetically engineered to combine direct viral oncolysis with FAP-expressing cancer-associated fibroblast killing by T cells via a FAP-targeted BiTE [129]. These two approaches aimed to enhance therapeutic efficacy by promoting both viral growth and immune reaction in tumor stroma elimination.

Another approach to enable viral growth in the extracellular matrix (ECM) is the use of ECM-targeted degradative enzymes, including chondroitinase ABC, which were recommended for inclusion in the herpes simplex virus genome, regulated by the IE4/5 promoter [130]. Other enzymes, collagenase and hyaluronidase, have also been investigated for use in promoting the delivery and diffusion of OVs within solid tumors [131].

Hypoxia is one of the important hallmarks of solid tumors resulting from a lack of vasculature in the core of the tumor tissue [132] . The physical barrier and dense extracellular matrix (ECM) in the tumor microenvironment (TME) also contribute to the development of the hypoxic condition, adding another dimension to the challenges that oncolytic virotherapy faces in solid tumors [132]. From one side, the hypoxic condition pushes toward an increase in angiogenesis and expression of pro-angiogenic factors such as vascular endothelial growth factor (VEGF) and interleukin-8 (IL-8). This in turn increases the permeability of the TME, and results in a temporary increase in the infiltration potential of the oncolytic virus (OV) progeny [132]. However, it also increases the infiltration of immune cells, making OV clearance and mounting an immune response more likely [132]. At the level of viral replication, hypoxia was reported to hinder the replication of several oncolytic viruses (OVs) that rely on the tumor cell translational machinery to produce viral proteins because it halts the hypoxic-induced cell cycle arrest at the G1 phase [133]. Several studies have reported the dual role of hypoxia in suppressing or enhancing the cytotoxicity and survival of OVs [134, 135]. However, in general, it is notable that most natural OVs with RNA-based genetic materials have been reported to thrive in hypoxic conditions, unlike DNA-based OVs, which are generally impaired under hypoxic conditions [136]. This could be attributed to the unique replication strategies of RNA viruses. Unlike DNA viruses, which often depend on the host cell’s nuclear machinery and S-phase progression for replication, RNA viruses typically replicate entirely within the cytoplasm using virus-encoded RNA-dependent RNA polymerases. Therefore, their replication is less reliant on the oxygen-sensitive transcriptional and cell cycle control mechanisms of the host. Moreover, many RNA viruses have evolved mechanisms to counteract the hypoxia-induced suppression of host protein synthesis. For example, some RNA viruses use internal ribosome entry sites (IRESs) or express viral proteins that maintain translation even under metabolic stress. This makes RNA OVs well adapted to infecting metabolically active or inactive cells found in hypoxic tumor cores. RNA OVs such as Newcastle disease virus (NDV) and reovirus not only tolerate hypoxia but actively exploit it; they downregulate hypoxia-inducible factor 1-alpha (HIF-1α), disrupting tumor adaptation pathways and facilitating both viral propagation and immune activation [62]. NDV can infect and kill hypoxic renal cell carcinoma cells by restoring von Hippel-Lindau (VHL) function and inducing interferon responses. Additionally, reovirus’s ability to inhibit HIF-1 expression further supports its potential in hypoxic tumors. For the same reasons mentioned above, hypoxic conditions suppress the replication of several DNA viruses, including adenovirus, herpes simplex virus type 1 (HSV-1), and SV40 [134]. While RNA OVs generally exhibit enhanced performance in hypoxic conditions, whereas DNA viruses are often impaired, certain viruses deviate from this trend due to unique replication strategies or host adaptations [134]. For example, vaccinia viruses (VACV) are one of the DNA viruses that show no change in their activity under normoxic or hypoxic conditions in different pancreatic cancer cell lines [137]. To overcome hypoxia-induced limitations and enhance tumor selectivity, several genetic engineering strategies have been employed to modify OVs, such as incorporating hypoxia-responsive elements (HREs), deleting viral genes to restrict replication to tumor cells, or arming viruses with immunostimulatory or antiangiogenic genes, as extensively reviewed in recent literature [132, 137].

Housing approximately 80% of the body’s total macrophage population, the liver serves as a major reservoir for Kupffer cells that act as initial filters, continuously clearing particles from the bloodstream. In addition, it constantly receives circulating myeloid cells, primarily blood monocytes that infiltrate the liver parenchyma [138]. Kupffer cells are a primary element of the hepatic immune microenvironment and are an important barrier to the systemic delivery and efficacy of OVs for hepatocellular carcinoma (HCC). Since they are phagocytic cells, the Kupffer cells rapidly sequester viral particles from the circulation, and up to 90% of adenovirus type 5 administered systemically has been reported to be internalized by them to a considerable degree, lowering virus titers in circulation and at the tumor site. Sequestration prevents effective transduction of hepatocytes and decreases the therapeutic efficacy of OVs. Choices such as pretreatment with warfarin to denude Kupffer cells or constructing chimeric adenoviruses with reduced binding avidity for coagulation factor X have been explored as ways of bypassing this hurdle [120]. This rapid sequestration by Kupffer cells exemplifies the innate immune clearance mechanisms described in Sect. "Host immune mechanisms limiting oncolytic virus persistence and activity", which can severely limit the bioavailability of systemically administered OVs. Conversely, their phagocytic activity may also promote antigen presentation and initiate anti‑tumor immune responses—illustrating the host’s dual role as both an adversary (antiviral immunity) and a potential partner (anti‑tumor immunity) in OV therapy. The tumor-associated macrophages TAMs, which constitute a vast percentage of the tumor environment and contribute as much as 50% of the mass in solid tumors, have a double-edged sword role in the case of oncolytic virotherapy (OVT) [139]. TAMs are polarized in HCC to M1 or M2 phenotypes. M1 macrophages have been reported to counteract OV therapy by recognizing and eliminating viral particles through their enhanced phagocytic activity, inducing an acute reduction in viral titers and a reduced therapeutic efficacy [140]. On the other hand, M2-like macrophages support tumor growth through exhibiting immunosuppressive functions such as VEGF-induced angiogenesis induction, extracellular matrix remodeling, and inhibition of T cells. An immunosuppressive function such as this has the potential to create a temporary refuge for OVs to proliferate and disseminate within the tumor. This advantage is, however, counteracted by the overall tumor-promoting function of M2 macrophages and suppression of anti-tumor immunity. OVs possess the ability to directly impact this dynamic by modulating the polarization of macrophages. Various studies have proven that OVs possess the potential to trigger the reprogramming of macrophages from the M2 to an M1 phenotype when they are recognized in response to the detection of PAMPs and DAMPs released during viral infection. These triggers activate macrophages and enhance the release of IFN-γ, triggering a pro-inflammatory microenvironment. This reprogramming has been observed with various OVs, including GL-ONC1, Ad5-D24-RGD [141], VSV [142], paramyxoviruses Measles [143], supporting the role of the OV in modulating the M1 to M2 ratio in the TME . Thus, while Kupffer cells pose great barriers to OV therapy of HCC due to their intrinsic viral scavenging, they are also potential candidates for therapeutic effect modulation via sufficient targeting or reprogramming [144, 145]. Similarly, the polarization of TAMs (M1 vs. M2) directly reflects the broader immune‑clearance versus immune‑activation dichotomy outlined in Sect. "Host immune mechanisms limiting oncolytic virus persistence and activity". Reprogramming TAMs toward an M1‑like phenotype can shift the balance from antiviral clearance toward sustained anti‑tumor immunity, thereby aligning the host’s immune response with therapeutic goals. Variability in HCC tumor origin, patient characteristics, tumor stage, and intrinsic tumor features all influence the heterogeneity in cellular composition and functional state of the TME (TME) and affect the OV tropism, making them regarded collectively as determining factors in the OV therapy response. They determine whether the TME exerts a supportive or suppressive role in tumor progression. Scientific evidence suggests that the route of OV administration, along with its compatibility with the TME, significantly influences transduction efficiency and tumor specificity. Physical features of tumors represent a significant barrier to intratumoral delivery of OVs [146]. More and more evidence indicates that the cellular milieu of the TME directly influences cancer pathogenesis. B-cell lymphomas describe the critical interaction between hematopoietic tumor cells and stromal cells. Some phenotypically and functionally altered cells in the bone marrow and the blood provide signals that suppress anti-tumor immunity and assist in tumor development [147, 148]. Adult T-cell leukemia is marked by a primarily immunosuppressive TME that mainly consists of cells that are CD4+ in nature [18].

These TME-specific challenges in HCC highlight the necessity for virus designs that exploit defective signaling or receptors in tumor cells, as detailed in the subsequent sections on HCC-targeted OVs and engineering tactics.

## Interactions between oncolytic viruses and hepatic viruses in HCC etiology

One major etiology of hepatocellular carcinoma (HCC) is chronic infection with hepatitis B virus (HBV) or hepatitis C virus (HCV), which promotes fibrosis, cirrhosis, and genomic instability leading to oncogenesis. These viral infections complicate oncolytic virus (OV) therapy, as OVs may interact with resident hepatic viruses in ways that influence efficacy, safety, and immune responses. For instance, in HCV-infected HCC, certain RNA-based OVs like reovirus (Reo) have shown dual benefits: They not only induce oncolysis but also trigger innate immune responses that suppress HCV replication, potentially reducing viral load and enhancing antitumor effects [149]. Similarly, vesicular stomatitis virus (VSV) exhibits increased sensitivity and lytic activity in HCV-infected HCC cells, suggesting a synergistic interaction where OV replication exploits HCV-altered cellular pathways [150]. In HBV-infected HCC, OVs such as vaccinia virus (e.g., JX-594) have been tested in clinical trials with HBV-positive patients, demonstrating partial responses without exacerbating HBV replication or causing severe hepatotoxicity [151]. However, HBV integrations into the host genome can promote oncogenic driver alterations, and OVs must navigate this altered microenvironment; preclinical data indicate that OVs like adenovirus may indirectly inhibit HBV through immune activation, but high HBV DNA levels could compete for cellular resources or trigger antiviral defenses that limit OV persistence [146]. Overall, while no major antagonistic interactions have been reported, OVs appear safe in HBV/HCV-co-infected patients, with potential for complementary antiviral effects, though careful monitoring for viral reactivation is essential, especially in immunosuppressed states induced by OV-mediated inflammation [152].

Conversely, harnessing hepatic viruses like HBV or HCV for cancer therapies poses significant challenges due to their inherent pathogenicity and association with HCC. Unlike naturally oncolytic viruses, HBV and HCV are not lytic by design and primarily cause chronic infection rather than direct cell killing. Engineering them as therapeutic vectors has been explored in limited contexts: For example, HBV core particles have been genetically modified to display tumor-targeting ligands (e.g., anti-HER2 single-chain antibodies), enabling targeted delivery of therapeutic payloads to HER2-positive cancers in preclinical models, with enhanced specificity and reduced off-target effects [153]. Similarly, recombinant HBV vectors have been used for gene therapy applications, leveraging their hepatotropism for liver-specific delivery, but safety concerns (e.g., insertional mutagenesis) limit their use as oncolytics [154]. HCV, being an RNA virus, is harder to engineer stably, and efforts have focused on pseudotyped vectors rather than direct oncolytic modification due to risks of persistent infection and fibrosis promotion [155]. While these approaches show promise for vector-based therapies (e.g., delivering suicide genes or immunomodulators), ethical and regulatory hurdles—stemming from their oncogenic potential—make them unlikely candidates for direct OV engineering. Instead, non-human RNA viruses (e.g., Newcastle disease virus or reovirus) are preferred for genetic modification in HCC treatment, as they can be safely attenuated and armed without the risks associated with HBV/HCV [156].

## Molecular mechanisms underlying HCC-selective oncolytic virotherapy

### Targeting via defective cell signaling pathways

From this point onward, we will focus on HCC, one of the most extensively studied cancers in the context of oncolytic virotherapy. Cancer cells often have dysregulated cellular pathways that set them apart from normal cells. These abnormal pathways provide opportunities for targeted therapy using genetically engineered or naturally occurring OVs, enabling them to selectively infect and destroy cancer cells. Several studies report this approach in hepatocellular carcinoma (HCC) using OVs. An overview of the engineering strategies used to target HCC with oncolytic viruses discussed in detail below, is summarized in Table 2. For example, VACV has been modified to target the EGFR-Ras signaling pathway, which is often dysregulated in HCC. This modification allows for the selective infection and replication of engineered strains like JX-594, which not only replicate within HCC cells but also target tumor-associated endothelial cells, highlighting JX-594’s potential as an OVs for HCC treatment [151].

**Table 2.** Summary of Oncolytic Virus (OV) engineering strategies and targeting mechanisms for Hepatocellular Carcinoma (HCC)

Targeting mechanism	Virus / strain example	Target / molecular trigger	Therapeutic action / result
Defective Cell Signaling	VACV (JX-594)	EGFR-Ras pathway	Selective infection; targets tumor-associated endothelial cells.
	Adenovirus (H101, ONYX-015)	p53/Rb deficiency (E1B deletion)	Limits replication to p53-deficient HCC cells.
	NDV (Lasota, Mukteswar)	Defective IFN signaling	Induces apoptosis and oncolysis in HCC; spares normal cells.
	ZD55-TRAIL / ZD55-Smac	Apoptotic pathways	Activates death receptors or removes apoptosis inhibitors (IAPs).
	GLV-1h68 / GLV-2b372	High nucleotide pools (TK deletion)	Requires cancer cell metabolism for DNA synthesis/replication.
Receptor-Mediated	Reovirus (Reo)	Sialic acid glycoproteins	Internalization via endocytosis leading to direct cell lysis.
	Coxsackievirus (V937)	ICAM-1	Cell lysis; synergistic with PD-1 checkpoint inhibitors.
	Measles Virus (MV-CEA)	CD46 receptor	Selective entry and syncytia formation in HCC cells.
Tumor-Specific Promoters	ZD55 / CV890	AFP Promoter	Restricts E1A/E1B expression to AFP-producing HCC cells.
	GD55	GOLPH2/GP73 Promoter	Selective replication in HCC; triggers anti-tumor immune response.
	OBP-301 / CNHK500	hTERT Promoter	Targets telomerase activity present in 90% of HCC cases.
	CNHK300	HRE + hTERT	Dual-targeting: telomerase activity and hypoxic tumor environment.
Engineering (Arming)	T-VEC / JX-594	GM-CSF expression	Recruits dendritic cells and activates cytotoxic T lymphocytes (CTLs).
	M032	IL-12 expression	Stimulates Th1 immune response and NK cell cytotoxicity.
	HSV-TK / CD	Suicide Genes	Converts non-toxic prodrugs (GCV/5-FC) into toxic metabolites (5-FU).
MicroRNA-Driven	LCSOV	miR-122 target sequences	Suppresses viral replication in normal liver cells (where miR-122 is high).

Similarly, adenoviral vectors have been designed to take advantage of the disruptions in the *Rb* and *p53* signaling pathways that are found in the majority of cases of HCC. Adenovirus types such as H101 and ONYX-015, for example, are genetically modified by deleting the E1B gene, which prevents E1B protein expression. This limits viral replication to p53-deficient cells, which is a typical state in HCC, thus conferring specificity [157, 158].

Another naturally occurring OV is the NDV, which relies on the relative insufficiency of interferon signaling that is typically dysregulated in HCC cells. Notably, NDV strains Lasota and Mukteswar have demonstrated outstanding cytotoxicity against HCC cells while sparing normal hepatocytes, illustrating the capability of such NDV strains to induce apoptosis and oncolysis in HCC treatment [159].

The adenovirus-based variants, such as ZD55-TRAIL and ZD55-Smac, were also designed to replicate preferentially in tumor cells, which frequently exhibit defective *p53* or *Rb* genes, a hallmark of many cancers [160, 161].

ZD55-TRAIL encodes the tumor necrosis factor apoptosis-inducing ligand, which induces apoptosis in cancer cells through activation of death receptors. On the other hand, ZD55-Smac acts by encoding the second mitochondria-derived activator of caspases (Smac) [162]. Smac removes the inhibitors of apoptosis proteins, thus naturally restoring the apoptosis signal and enhancing the apoptotic effect in cancer cells [163, 164]. Furthermore, ZD55-IFN-β is designed to express interferon-β, which modulates the TME by enhancing immune-mediated cytotoxicity and blocking angiogenic signaling pathways [165].

The VACV family also capitalizes on essential cellular mechanisms involved in DNA synthesis and repair, particularly in cancer cells, which naturally have high nucleotide pools due to abnormally regulated metabolic pathways. Strains like GLV-1h68 and GLV-2b372 use thymidine kinase (TK) deletions to prevent viral replication in host cells but replicate efficiently in cancer cells, which provide adequate nucleotide pools due to dysregulated metabolism. In addition, they can use inflammatory pathways and vascular remodeling pathways to further eliminate the tumor vasculature and enhance anti-tumor immune response, leading to increased overall therapeutic effectiveness [150, 166]. Through the advanced genetic engineering and natural selectivity of OVs, highly specific HCC treatments have been developed with minimal damage to normal tissue.

### Receptor-mediated targeting

Neoplastic cells typically express a distinct set of receptors, which are selectively used as the sites of entry into the cell by OVs. Attaching to such overexpressed or cancer-specific receptors, OVs enter into cancer cells but with minimal contact with normal cells. Several overexpressed receptors on hepatocellular carcinoma (HCC) cells have been identified and exploited to design receptor-specific OV therapies. For example, oncolytic reovirus (Reo) can target liver cells through binding to sialic acid-containing glycoproteins on the cell surface before being internalized by the cell through endocytosis. Once reovirus is internalized, it begins to replicate its genome, leading to direct cell lysis as well as initiating an innate immune response [149].

Another OV that enters HCC cells through receptor-mediated entry is V937, a coxsackievirus A21 prototype virus derivative. V937 enters cells by binding to the adhesion molecule ICAM-1, which is abundantly expressed on the surface of various cancer cells, including HCC, and then replicates, ultimately leading to cell lysis. This activity is normally accompanied by eliciting an immune response. In addition, the combination of V937 with immune system checkpoint inhibitors, such as PD-1 blockade, has been shown to effectively target immunologically inert tumors [152, 167].

Measles vaccine virus, such as MV-CEA strain, is also an OV candidate that enters various cancers, including HCC, through the use of the CD46 receptor [168]. The measles virus infects the cancer cells and multiplies within them, killing the cells and releasing viral particles to infect neighboring tumor cells. The immune response that the measles virus creates is also enhanced in the identification and killing of tumor cells by the immune system [167].

### Targeting driven by tumor-specific promoter

Promoter-based targeting is another approach that uses tissue-specific gene regulatory elements to regulate viral replication, thus improving OV therapy safety by restricting its activity to target cancer cells.

The *AFP* promoter has been extensively utilized in this context because AFP is expressed at high levels in most HCC cells but exhibits low activity in normal adult tissues [169].

ZD55, a modified adenovirus serotype 5 (Ad5), incorporates an E1B-55K deletion and uses the alpha-fetoprotein (AFP) promoter, allowing selective replication in AFP-producing cells [170]. Another example is CV890, which employs a dual regulatory system using both the AFP promoter and enhancer elements to control E1A expression, a gene essential for initiating the adenoviral replication cycle [171].

Additionally, the construction of AAV recombinants carrying the herpes simplex virus thymidine kinase (HSV-TK) gene, under the transcriptional control of the AFP enhancer and albumin promoter, has been reported [172]. This construct allows the selective expression of TK gene in hepatocellular carcinoma. The TK enzyme phosphorylates the antiviral drug ganciclovir into a toxic metabolite, and it is this toxic metabolite that induces apoptosis in AFP-positive cancer cells but not AFP-negative and non-tumor cells [173].

Another example of promoter-based targeting is *GP73/GOLPH2*. It is a Golgi membrane protein that is overexpressed in HCC tissues compared to normal liver tissues, enabling use of the GOLPH2 promoter for selective replication in HCC cells.

The recombinant oncolytic adenovirus GD55 expresses the E1A gene under the transcriptional control of the GOLPH2 promoter. This allows the virus to achieve selective replication in HCC [174, 175]. Studies have shown that GD55 significantly hindered tumor growth during HCC xenograft [176, 177]. Moreover, the GOLPH2 promoter’s activity has been linked to the induction of immune responses against the tumor, enhancing the overall antitumor response [178].

The hTERT promoter is another example of this approach. hTERT is the catalytic subunit of telomerase, an enzyme important for maintaining telomere length and cellular immortality. Telomerase activity is silenced in most of the somatic cells, but it is expressed in about 90% of cancers, making the hTERT promoter one of the best candidates for therapeutic targeting in cancer [179]. Adenovirus serotype 5 (Ad5)-derived CNHK500 was developed using the hTERT promoter for the regulation of E1A gene expression, taking advantage of the highly expressed telomerase activity that occurs in about 85-90% of HCC cases, hence it is potentially more reliable than the targeting mechanism used by AFP [180]. CNHK300 was built on this strategy by combining Hypoxia-Responsive Elements (HREs) with the hTERT promoter, a dual-targeting mechanism that takes advantage of both elevated telomerase activity and the hypoxic TME of HCC tumors [181]. The combinatorial strategy significantly improved cancer cell-selective viral replication with minimal effect on normal tissue. Telomelysin (OBP-301) is also one of the most clinically advanced applications of hTERT promoter-driven targeting [182]. It achieves striking cancer specificity through the control of both E1A and E1B expression through the hTERT promoter. Clinical trials have affirmed its safety profile and promising antitumor activity, showing that this approach can have significant therapeutic potential [183].

### Targeting by engineered oncolytic virus

OVs are engineered to express novel molecules or proteins not naturally present in the host cells to enhance the immune response or expedite the killing of the infected host cells.

For example, talimogene laherparepvec (T-VEC), a herpes simplex virus type 1 is modified to express granulocyte-macrophage colony-stimulating factor GM-CSF. T-VEC can induce lytic cell death in infected cancer cells, leading to the release of tumor-associated antigens and danger signals that activate the immune system [184]. It is complemented by the expression of GM-CSF, which recruits dendritic cells into the TME, leading to the activation of cytotoxic T lymphocytes (CTLs) [185]. This dual action makes T-VEC an appropriate candidate for utilization in combination with immune checkpoint inhibitors [186]. Similarly, JX-594 (or OncoVEX) is an oncolytic vaccinia virus, which has been genetically modified to secrete GM-CSF. This virus has been designed to selectively infect and replicate in cancer cells that are expressing specific markers of malignancy, i.e., alpha-fetoprotein (AFP) in the HCC. The mechanism of action of JX-594 involves direct oncolysis and the induction of a systemic immune response as well. Upon infection of HCC cells, JX-594 replicates and causes cell lysis, thereby releasing tumor antigens that have the ability to induce an immune response [187, 188]. Another example is M032, an oncolytic herpes simplex virus engineered to express interleukin-12 (IL-12), a potent cytokine that enhances antitumor immunity [187]. M032 infection induces cell lysis, which in turn releases IL-12 to act in an autocrine and paracrine manner on the activation of immune cells within the TME [71]. IL-12 stimulates the differentiation of naive T cells to effector T cells, enhancing the cytotoxicity against HCCs by NK cells, thus enhancing the immune response against HCC.

Another method, "suicide gene therapy," involves introducing genes from viruses or bacteria and giving the cell the ability to convert nontoxic prodrugs into cytotoxic compounds or to generate toxic proteins specifically in the tumor cells without harming normal cells [189]. Two of the most researched suicide gene systems are herpes simplex virus thymidine kinase (HSV-TK)/ganciclovir (GCV) and cytosine deaminase (CD)/5-fluorocytosine (5-FC) systems [190, 191]. The HSV-TK/GCV system involves the expression of the HSV-TK enzyme in cancer cells, which phosphorylates the prodrug GCV into a cytotoxic metabolite that induces apoptosis. This toxic metabolite diffuses into adjacent tumor cells, amplifying the therapeutic impact. The CD/5-FC system follows a similar approach by using the bacterial CD enzyme, which does not have its counterpart in mammalian cells, to convert 5-FC into 5-fluorouracil (5-FU), an active anticancer agent. Of note, tumor specificity with minimum side effects has been shown for the CD/5-FC system [191].

### Targeting driven by microRNA

miRNA-based targeting takes advantage of the differential expression of microRNAs between normal and malignant liver cells, especially utilizing liver-specific miRNAs whose expression is strongly downregulated in HCC. This approach is based on the genetic modification of viruses in a way that miRNA target sequences are inserted into essential viral genes, thus generating a kind of molecular switch that makes viral replication dependent on cellular miRNA profiles. Therefore, cells abundant in the corresponding miRNA will degrade viral transcripts and suppress replication. The most popular target of this therapeutic approach so far is miR-122, which represents about 70% of liver miRNAs and is strongly downregulated in HCC [192]. A study by Fu et al. constructed a liver-cancer specific oncolytic virus (LCSOV) containing an apoE promoter with target sequences for miR-122, which selectively targeted HCCs without affecting normal liver tissue [193]. This idea has been further studied by other research groups in which additional liver-related miRNAs, such as miR-199a and miR-124a, have been incorporated, further increasing the specificity of the OV to HCCs [194]. This strategy provides multiple routes of tumor selectivity, improves safety profile through miRNA-responsive elements, and reduces systemic toxicity associated with conventional oncolytic therapies [192].

By leveraging these targeting mechanisms, OVs can achieve greater precision in HCC; yet, broader therapeutic success requires integrating them with advanced delivery methods to ensure effective viral spread, as examined next.

## HCC organoids to study OV therapy

The cancer microenvironment is complex and comprises a wide range of parenchymal and non-parenchymal cells, leading to heterogeneity at the level of the tumor itself and between different tumors. To address the heterogeneity of the cancer microenvironment and to understand cancer biology, several preclinical models have been established. Preclinical models can be divided into *in vitro* models, like cancer cell lines and organoids, and *in vivo* models, like animal models. Organoids are promising, yet incomplete, preclinical platforms that recapitulate key aspects of tumor architecture and heterogeneity for studying cancer behavior and oncolytic virus (OV) therapy [170]. Organoids present a 3D culture model that provides the cells with a complex microenvironment and retains tumor heterogeneity. Organoids are known to self-organize into a structure that mimics and recapitulates the original tissue from which they are derived [195]. In a comparative study, it was noted that organoids of the breast tumor were concordant with the host tumor in DNA copy number, in contrast to cell line-derived models [195]. Organoids grow rapidly and avoid the interspecies interaction issues inherent in xenograft models [196], and therefore can be considered a highly relevant model for cancer research [197, 197]. The use of organoids and three-dimensional culture systems in oncolytic virotherapy research has transformed our understanding of virus-tumor interactions and therapeutic outcomes. Such advanced models have unveiled important insights that were masked by the limitations of traditional two-dimensional cultures. . These models have highlighted the effects of cellular heterogeneity, interstitial matrix composition, and tumor architecture on viral dissemination and therapeutic response. They have also provided critical insight into the mechanisms of viral resistance and therapeutic failure that allow for more informed approaches to the design and delivery strategies of Ovs [198].

A recent preclinical study evaluated the oncolytic activity of the coxsackievirus A21–based oncolytic virus V937 in hepatocellular carcinoma (HCC) using tumor cell lines and patient-derived organoids. Organoids were generated from surgically resected HCC tissues through enzymatic dissociation, embedded in Matrigel, and cultured in organoid-specific media, with histopathological analyses confirming their malignant origin. Treatment with V937, alone or in combination with the PD-1 inhibitor pembrolizumab, resulted in enhanced oncolysis, increased IFN-γ production, and upregulation of ICAM-1 expression on HCC cells, indicating improved immune activation. Despite these promising findings, the authors noted limitations related to the simplified immune co-culture system, which did not fully recapitulate the complexity of the tumor microenvironment, and emphasized the need to evaluate a broader spectrum of cytokines and chemokines beyond IFN-γ [167].

In another study, Samson et al., employed several models, including primary human liver tissues, HCC cell lines, and *in vivo* xenograft mouse models, to explore the oncolytic activity of reoviruses in combination with antiviral and anti-tumor agents in HCC. The study also explored whether innate immune responses induced by Reo could effectively target both HCC cells and the oncogenic viruses such as HCV [149]. The findings demonstrated that Reo effectively inhibits HCV replication through IFN responses, lowers tumor growth in HCC models, and increases NK cell activity, all of which contribute to antitumor efficacy. Managing toxicity in immunocompromised mice and ensuring that immune-driven effects translate effectively to human clinical settings remains a major challenge [149].

Despite their advantages, current organoid models have inherent limitations that must be considered. They typically lack a fully functional, systemic immune system, including circulating lymphocytes, myeloid cells, and lymphoid structures, which restricts the study of adaptive immune responses and systemic immune surveillance critical to OV therapy [199]. Furthermore, the absence of vascularization and fluid flow in static organoid cultures impedes the investigation of hemodynamic OV delivery, immune cell trafficking, and metastatic processes [200]. These simplifications can overestimate viral penetration and underestimate immune-mediated clearance, limiting their predictive value for clinical translation.

To address these gaps, emerging strategies are enhancing organoid complexity. Co-culture systems integrating autologous immune cells (e.g., peripheral blood mononuclear cells, tumor-infiltrating lymphocytes, or engineered CAR-T cells) enable the study of immune–OV–tumor interactions in a human-specific context [201]. Microfluidic organ-on-a-chip platforms that incorporate endothelial cells and perfusion can model vascular delivery, interstitial pressure, and hypoxia gradients, key determinants of OV biodistribution [202]. Additionally, incorporating patient-derived cancer-associated fibroblasts (CAFs) and ECM components better recapitulates the fibrotic barriers characteristic of HCC [203].

Organoids are thus best suited to answer specific, compartmentalized questions in OV development: (1) intrinsic oncolytic potency, viral tropism, and replication kinetics in 3D tumor architectures; (2) patient-specific responses and heterogeneity using PDOs; (3) local immune modulation (e.g., macrophage polarization, cytokine secretion) in co-culture setups; and (4) high-throughput screening of OV–drug combinations. While they cannot replace in vivo models for systemic immunity studies, they provide an essential bridge between 2D cultures and animal models, enabling mechanistic, human-relevant preclinical insights with controlled complexity.

## Biological barriers and technological advances in OV delivery

Accumulated data on OV clinical trials show that despite the promising results in the lab, there are no prominent signs of success in clinical cases [204]. This is due to the hurdles or ‘traps’ that OVs must overcome to achieve optimal infection of cancer cells [205]. The potential traps discussed below are preexisitng immunity, heterogeneity of the tumor environment, and the route of delivery (Fig. 3). Figure 3 synthesizes the major delivery barriers within the HCC microenvironment, categorizing them into physical/structural hurdles (e.g., fibrotic ECM, interstitial pressure), immune-mediated clearance (e.g., by Kupffer cells, immunosuppressive myeloid cells), and stromal shielding, thereby visualizing the multifaceted challenge facing systemic OV delivery. OVs improve the anti-tumor immune response, surpassing the prevalent immunosuppression and modifying the TME by activating the immune system [136]. OVs increase immune cell infiltration, converting ‘cold’ tumors with low immune activity into ‘hot’ tumors with abundant immune cells by activating adaptive anticancer immunity [206]. The immune response equation determines whether or not the viral therapy is effective. The immunogenicity of OVs is essential to elicit an effective anti-tumor immune response. Macrophages, as key initiators of this response, can bridge innate and adaptive immunity and contribute to both anti-pathogen defense and anti-tumor activity. However, the same immune response may also act against the virus itself, thereby limiting treatment effectiveness [136]. During the antiviral immune response, OVs in circulation are recognized and neutralized by antibodies, while the infected cells are targeted by cytotoxic CD8+ T cells that are virus-specific. The effects of preexisitng immunity are predominantly due to a humoral response against the viral infection, hence a seroprevalence screening is usually required before starting a clinical trial [207]. In light of this, the research focus has been on the effect of preexisitng immunity on the first phases of infection of the tumor by OVs, including infection, spread in circulation, and dissemination to other tumors [207]. The occurrence of viral neutralization by the high amounts of preexisitng neutralizing antibodies against certain types of OVs can be classified as the biggest hurdle when it comes to effective treatment with OVs [208]. A deeper understanding of adaptive immune dynamics, including memory T and B cell responses, is essential for engineering OVs that can evade or repurpose pre-existing antiviral immunity [209]. This impedes the OVs from replicating and thereby limits the lysis of cancer cells [108]. One of the biggest effects of preexisitng immunity on OVs is the prevention of the dissemination of the virus, which is crucial for the effectiveness of the viral therapy when it comes to targeting multiple tumors [207]. The resulting overreaction of an inadequately regulated immune response can cause damage to healthy tissues and infected cells, eliminating them and neutralizing the virus, thus compromising the effectiveness of the OVs. The epidemiological aspects of the parental virus of OVs are reflected in the seroprevalence of neutralizing antibodies [105]. This emphasizes the value of design optimization through a deep analysis of the factors influencing the immune reaction, namely, the presence of antigen-specific T-cells [105]. The consensus is that the presence of preexisitng immunity enhances viral clearance, which limits the window of action required for a therapeutic effect [207]. As an attempt to prevent the interference of preexisitng immunity, some studies are opting for the use of rare serotypes of OVs [208]. Alternative strategies to circumvent this limitation include capsid protein modification and physical protection of viral particles. Some strategies use chemical conjugation of the capsid with polymers that protect the virus but hinder its infectivity [208, 210]. Using albumin as a drug carrier has been a recent research trend; besides reducing clearance, it accumulates in solid tumors, providing a tumor-targeting effect. One study utilized these factors to create an albumin-binding adenovirus as protection from neutralizing antibodies. They did that by adding the albumin-binding domain 3 to the HVR1 of the adenovirus hexon, and their results showed an increase in the OV plasma half-life [210]. Researchers have tried to cloak the OVs with mesenchymal stromal cells owing to their tumor-homing abilities and low immunogenicity; this strategy allows for efficient delivery and reduced neutralization and clearance [211].

Interestingly, NDV is one of the OVs that has demonstrated that, although preexisitng antiviral immunity may limit viral replication, the heightened immune response can still produce successful outcomes in animal models of cancer treatment [108]. More evidence suggests that the effector responses due to preexisitng immunity can contribute to therapeutic efficacy when leveraged and redirected toward the tumor using bispecific molecules [212]. A study utilizing this phenomenon designed a bispecific adaptor molecule with an Adenovirus 5 epitope and a domain that binds to polysialic acid, which is a molecule that allows surface adhesion associated with a range of cancers. Their results were optimistic, showing an improved survival rate in mice modeling colon carcinomas, lung carcinomas, and melanomas [212].

The heterogeneous environment of tumors poses another hurdle that the virus must overcome, including fibrosis, ECM, and hydrostatic pressure, all of which can act as a strong barrier preventing OVs from infecting the cancer cells [108].

The use of intratumoral injections directly into the tumor site should provide a more efficient delivery by avoiding the neutralization of the virus; however, this is a technically advanced method that requires interventional radiology, depending on the location of the tumor. In addition to this, the virus can still face the hurdle of preexisitng immunity when traveling to other nodules, even when it is injected intratumorally. Furthermore, if a tumor has multiple nodules, the OVs are required to be administered systemically to provide a higher chance of infectivity [213]. It has been noted that the production of antibodies to neutralize the virus appears to be partially dependent on the route of administration as well. Intravenous administration allows the targeting of the primary tumor and the disseminated tumors by the OVs [214]. It has been reported that local injection of OVs can be coupled with immune agents, and the intratumoral injection of OVs can also be used as a pretreatment to increase tumor-infiltrating lymphocytes that can be used in adoptive cell therapy [215].

## Clinical translation: lessons from success and failure

The clinical translation of oncolytic viruses provides critical insights that directly inform the rational design of next-generation platforms

### The approved vanguards: proof of concept and its inherent limitations

The regulatory approvals of talimogene laherparepvec (T-VEC) for melanoma and Delytact (G47Δ) for glioblastoma represent watershed moments, validating core principles of OV therapy. T-VEC’s success demonstrates that local intratumoral delivery of a GM-CSF-armed virus can generate systemic, abscopal responses in a proportion of patients, cementing the "in situ vaccine" paradigm [88, 184]. Similarly, Delytact’s conditional approval in Japan for recurrent glioblastoma confirms that neurotropic viruses can be safely engineered for use in the CNS, a critical safety benchmark [89]. However, these successes are contextually bounded. Their efficacy is primarily demonstrated in locally advanced, injectable tumors, highlighting that the primary barrier for broader application remains effective systemic delivery and penetration into visceral or multiple metastatic sites. Furthermore, response rates as monotherapy are modest, underscoring the necessity for combination strategies and more potent viral engineering.

### The HCC clinical landscape: a crucible for solid tumor virotherapy

HCC trials have served as a stringent testing ground, revealing both promise and profound challenges. The oncolytic vaccinia virus JX-594 (Pexa-Vec) demonstrated in Phase II trials that it could be safely administered via intrahepatic arterial infusion, with evidence of dose-dependent replication in tumor tissue and induction of antitumor immunity [151, 187]. Yet, its Phase III trial (PHOCUS) in advanced HCC failed to meet its primary overall survival endpoint when combined with sorafenib. This failure is instructive: it suggests that in late-stage, heavily pretreated HCC patients, a single-armed virus may be insufficient to overcome the profoundly immunosuppressive and fibrotic TME. Similarly, trials of oncolytic adenoviruses (e.g., H101/Oncorine) have shown safety but limited systemic efficacy, often constrained by high seroprevalence of neutralizing antibodies and rapid hepatic clearance [157].

These trials collectively emphasize several translational gaps for HCC: 1) Route matters: Intra-tumoral or intra-arterial delivery may bypass some systemic barriers but is not feasible for disseminated disease. 2) Timing is critical: OVs may be more effective in earlier-stage disease or as adjuvant therapy, before the establishment of a fully immunosuppressive TME. 3) Biomarkers are absent: We lack predictive biomarkers (e.g., baseline immune infiltrate, interferon signature, or ECM composition) to identify patients most likely to respond, leading to trials in unselected, often refractory populations.

### Instructive failures: the critical role of trial analysis

The failure of the Phase II trial evaluating Senecavirus A (NTX-010) in small-cell lung cancer (SCLC) following chemotherapy is a paradigm-instructive setback. SCLC possesses a dense, proliferative, and immunologically "cold" TME. The trial’s negative outcome likely reflects multiple, now-recognized hurdles: the virus may have been unable to achieve sufficient intratumoral replication and spread against rapid tumor turnover; the profoundly immunosuppressive milieu may have quenched the immunogenic response; and heavily pre-treated patients may have had limited immune reserves to engage [216]. This failure underscores that not all viruses are suitable for all tumor types and that the TME must be matched with an OV platform possessing complementary capabilities (e.g., hypoxia tolerance, stromal degradation).

### Synthesis: key translational takeaways for next-generation design

Delivery is Paramount: Clinical success has been largely confined to locally delivered viruses. For OVs to become a systemic therapy, engineered solutions to immune clearance, hepatic sequestration, and poor tumor penetration are non-negotiable. Monotherapy is Likely Insufficient for Advanced Cancers: Durable responses typically require combination with modalities that modulate the TME (checkpoint inhibitors) or synergistically kill tumor cells (chemotherapy, targeted therapy). The "Cold" TME is a Major Cause of Failure: Viruses must be actively armed or combined with agents that reverse immunosuppression, recruit effector cells, and degrade physical barriers. The Field Lacks Predictive Biomarkers: Future trials must incorporate robust translational components to identify which patients, based on tumor genetics and immune contexture, are most likely to benefit from specific OV platforms. These clinical insights validate the progress in OV therapy but also reveal persistent gaps, informing future directions in engineering, combination therapies, and personalized approaches to fully realize OVs’ potential [217].

## Future perspectives in OV research

Next-generation OVs outside of current limitations are being facilitated by advances in immuno-oncology and synthetic biology technology. Next-generation OVs are being revealed by technological advances in immuno-oncology and synthetic biology that move beyond current limitations as well as therapy-related potency [218]. Precision medicine approaches, e.g., patient-specific OVs tailored to the patient’s tumor genome and immune microenvironment, are a promising frontier of future OV research. The marriage of immune oncology and synthetic biology is expected to lead to advanced strategies to overcome resistance as well as augmentations in anti-cancer immune responses [219]. Emerging efforts concentrate on creating OVs that utilize unique tumor indicators, mutational profiles and immune evasion mechanisms for patient-specific therapy [220]. The future directions of oncolytic virotherapy will also include multi-armed viruses, which simultaneously target the cancer cell itself, as well as its immunosuppressive microenvironment, according to the principles of precision oncology [221]. Combination of OVs with PD-1/PD-L1 inhibitors has been demonstrated to transform immunologically ‘cool’ tumors into ‘hot’ ones, with enhanced T-cell infiltration and enhanced therapeutic effects.

Preclinical and early-phase clinical trials suggest that combining OVs with immune checkpoint blockade can overcome resistance to monotherapy in some settings, potentially transforming immunologically ‘cold’ tumors into ‘hot’ ones [222] However, clinical efficacy remains variable and is often confined to a subset of patients. This mixed evidence underscores that such combinations are not universally effective and highlights the need to identify the determinants of response, such as baseline immune infiltration, viral replication efficiency, and the specific immunogenic profile of the tumor, to guide personalized therapeutic strategies. The integration of OVs with conventional therapies (chemotherapy, radiotherapy) and emerging immunotherapies (CAR-T, checkpoint inhibitors) is redefining combination strategies to maximize tumor debulking and immune activation [223].

Genetically modifying OVs to secrete cytokines (i.e., IL-12, GM-CSF) or immune activators augments their two-way functionality as direct tumor-lytic treatment and in situ cancer vaccines. Equipping OVs with immune-modulatory transgenes renders them equally powerful immunotherapeutic agents able to restructure the TME [126]. Local delivery of PD-1/PD-L1 blockers by genetically modified OVs triggers intratumoral immune activation with less systemic toxicity than antibody-based therapy [224].

Nanoparticle-encapsulated OVs have enhanced pharmacokinetics through viral protection from neutralizing antibodies and enhanced tumor-specific accumulation. In addition, cell-based carriers, such as mesenchymal stem cells (MSCs) or immune cells, are a ‘Trojan horse’ approach to escape immune elimination and penetrate tumors more effectively [225]. Moreover, extracellular vesicles (EVs) engineered to encapsulate OVs present a novel stealth delivery mechanism, avoiding immune detection and improving biodistribution. Innovations in biomaterial-based carriers (e.g., hydrogels, nanoparticles) are overcoming extracellular matrix (ECM) barriers in solid tumors, enhancing intratumoral viral spread [226]. Stem cell-mediated OV delivery not only protects the virus from systemic neutralization but also homes to tumor sites via inherent tropism, improving therapeutic precision. Physical constraints such as dense stroma and high interstitial pressure in solid tumors are being overcome using sophisticated delivery platforms such as tumor-penetrating peptides and ECM-degrading enzymes [227].

Nanocarriers with tumor-targeting ligands (e.g., RGD peptides) improve OV delivery to tumors and reduce off-target toxicities. OVs armed with ECM-modulating drugs (e.g., hyaluronidase) promote viral dissemination in fibrotic tumors, overcoming an important limitation of virotherapy [155].

The second-generation OV delivery systems employ immune cell hitch-hiking (e.g., NK and T cells) to improve tumor trafficking while minimizing antiviral immune responses. Exosome-coated OVs traveled deeper into tumors and circled for a longer period, indicating a refinement of systemic administration for metastatic cancer [156]. Recently, advanced 3D tumor organoids and microphysiological systems now enable high-fidelity human tumor heterogeneity, stromal interactions, and oncolytic viral spread models to bridge the gap between traditional in vitro research and therapeutic outcomes. In pre-experimental validation, AI-driven computational methods are being incorporated into OV design platforms for forecasting suitable genetic alterations, tropism, and immune evasion mechanisms [228]. Ex vivo 3D tumor models derived from patient samples are a simulation of the physical and immune barriers seen in the tumor environment and hence offer a revolutionary platform to investigate OV penetration, replication kinetics, and mechanisms of resistance. These strategies deliver real-time monitoring of immune cell infiltration and cytokine signatures after OV infection, creating data on bystander effects and combinatorial immunotherapy potential [229]. In silico simulation of OV-tumor interactions under spatial limitations and drug-resistant targets predicts therapy failure and allows the design of optimized viruses with increased fitness. Computational modeling of viral dissemination in 3D tumor models defines critical thresholds in interstitial pressure and ECM density that constrain OV performance, calling for delivery optimization [230]. 3D coculture systems with stromal components (e.g., adipocytes, fibroblasts) mimic the immunosuppressive conditions encountered in solid tumors, illustrating how OVs alter the TME to overcome resistance. Furthermore, 3D model fluorescent labeling of OVs quantitates viral tropism and replication competence in real-time, allowing preclinical screening [231]. Organoid-based OV testing systems outperform monolayer cultures in predicting clinical responses because they preserve tumor-stroma communication and hypoxic gradients. Future OV development will include machine learning to analyze multi-omics data from 3D models, allowing personalized viral selection based on tumor molecular profiles [232]

The clinical translation of oncolytic virotherapy, particularly for refractory tumors like glioblastoma, pancreatic carcinoma, and HCC, necessitates stringent long-term safety assessments to address risks such as viral recombination with wild-type or latent genomes, off-target damage, and unpredicted immune activation [108, 233]. Systemic administration, while needed for metastatic disease, carries the inherent risk of triggering antiviral immune responses that can limit efficacy, underscoring the necessity for stealth delivery strategies [108]. Furthermore, the tight fibrotic microenvironments of tumors like pancreatic cancer and glioblastoma require OVs engineered to maximize penetration while minimizing risks of organ-specific toxicities such as pancreatitis and neurotoxicity [108]. Regulatory frameworks must evolve to facilitate the unusual pharmacokinetics and biodistribution of OVs, especially with new delivery vehicles (e.g., cell carriers, nanoparticles) and engineered vectors [108]. Standardizing safety evaluations across tumor types and virus platforms, managing the challenges of personalized OV production, and monitoring critical factors like viral shedding and environmental persistence are essential for advancing the field [108, 233].

## Conclusions

Hepatocellular carcinoma remains a formidable clinical challenge, characterized by an immunosuppressive and fibrotic tumor microenvironment that limits conventional therapies. Oncolytic viruses have emerged as a promising multi-faceted strategy, combining direct tumor lysis with the induction of systemic anti-tumor immunity. This review highlights that the most effective OV strategies for HCC are those engineered to overcome the unique barriers of the liver TME, through genetic modifications that enhance tumor selectivity (e.g., via HCC-specific promoters such as AFP or hTERT), arm viruses with immunomodulatory transgenes (e.g., GM-CSF, IL-12, or checkpoint inhibitors), and exploit dysregulated signaling pathways (e.g., p53/Rb defects or activated Ras).

Despite considerable progress, significant hurdles persist. Immune-mediated clearance, by Kupffer cells, neutralizing antibodies, and antiviral cytokines, remains a major bottleneck for systemic delivery. The dense, fibrotic extracellular matrix of HCC limits viral penetration and spread, while tumor heterogeneity and hypoxic cores further constrain therapeutic efficacy. Translational success will depend not only on smarter viral engineering but also on innovative delivery solutions, such as cell-based carriers, nanoparticle encapsulation, and ECM-modulating adjuvants.

Moving forward, the integration of OVs with other modalities—immune checkpoint inhibitors, targeted therapies, locoregional treatments, and conventional chemotherapy—will be critical to unlock synergistic responses. Advanced preclinical models, including immune-competent organoids and organ-on-a-chip systems, will better predict clinical outcomes and personalize therapeutic regimens. Furthermore, emerging technologies in synthetic biology, AI-driven design, and real-time biosensing promise to accelerate the development of next-generation OVs tailored to individual patient profiles.

In summary, oncolytic virotherapy stands at a pivotal juncture. By continuing to bridge insights from virology, immunology, and bioengineering, the field is poised to transform OVs from experimental agents into cornerstone immunotherapies for HCC and other solid tumors. The journey from bench to bedside will require persistent innovation, thoughtful combinatorial approaches, and rigorous clinical validation—ultimately offering new hope for patients with historically untreatable cancers.

## Supplementary Information

Additional file 1.

Additional file 2.

## Data Availability

All the data presented in this review are available and presented in the text.

## Abbreviations

4-1BBL: 4-1BB ligand (CD137 ligand)
5-FC: 5-Fluorocytosine
5-FU: 5-Fluorouracil
AdV: Adenovirus
AFP: Alpha-fetoprotein
AKT: Protein kinase B
APCs: Antigen-presenting cells
BCG: Bacillus calmette–guérin
BiTE: Bispecific T-cell engager
CALR: Calreticulin
CAFs: Cancer-associated fibroblasts
CAR-T: Chimeric antigen receptor T-cell
CD: Cytosine deaminase
CD40L: CD40 ligand
CD47: Cluster of differentiation 47
CD155: Poliovirus receptor
CDKN2A: Cell cycle-dependent protein kinase inhibitor 2A
CEA: Carcinoembryonic antigen
cGAS: Cyclic GMP-AMP synthase
CTLs: Cytotoxic T lymphocytes
CTLA-4: Cytotoxic T-lymphocyte–associated protein 4
CXCL9: C-X-C motif chemokine ligand 9
CXCL10: C-X-C motif chemokine ligand 10
DAMPs: Damage-associated molecular patterns
DCs: Dendritic cells
ECM: Extracellular matrix
EGFR: Epidermal growth factor receptor
EMA: European medicines agency
E2F-1 (E2F1): E2F transcription factor 1
FAP: Fibroblast activation protein
FDA: Food and drug administration
FOLFOX: Folinic acid, fluorouracil, and oxaliplatin
GCV: Ganciclovir
GD2: Disialoganglioside
GFP: Green fluorescent protein
GM-CSF: Granulocyte-macrophage colony-stimulating factor
GP73: Golgi protein 73
HCC: Hepatocellular carcinoma
HCMV: Human cytomegalovirus
HG52: Herpes simplex virus type 2 strain HG52
HIF-1α: Hypoxia-inducible factor 1-alpha
HREs: Hypoxia-responsive elements
HSCs: Hepatic stellate cells
HPV: Human papillomavirus
HSV-1: Herpes simplex virus type 1
HSV-TK: Herpes simplex virus thymidine kinase
H-1PV: Parvovirus H-1
ICAM-1: Intercellular adhesion molecule 1
ICD: Immunogenic cell death
IFN: Interferon
IFN-α: Interferon-alpha
IFN-β: Interferon-beta
IFN-γ: Interferon-gamma
IL: Interleukin
IL-1: Interleukin-1
IL-2: Interleukin-2
IL-6: Interleukin-6
IL-12: Interleukin-12
IL-15: Interleukin-15
IL-24: Interleukin-24
JAM: Junction adhesion molecule
JAK-STAT: Janus kinase–signal transducer and activator of transcription
lacZ: β-Galactosidase reporter gene
LCMV: Lymphocytic choriomeningitis virus
LDL-R: Low-density lipoprotein receptor
LFA-3: Lymphocyte function-associated antigen 3
LOX: Lysyl oxidase
MAGE-A3: Melanoma-associated antigen A3
MHC: Major histocompatibility complex
MMPs: Matrix metalloproteinases
MV-CEA: Measles virus–carcinoembryonic antigen
MYXV: Myxoma virus
NDV: Newcastle disease virus
NF-κB: Nuclear factor-kappa B
NIS: Sodium Iodide symporter
NK: Natural killer
NMIBC: Non–muscle-invasive bladder cancer
NS1: Non-structural protein 1
NSCs: Neural stem cells
orfC: Open reading frame C
OVs: Oncolytic viruses
OVT: Oncolytic virotherapy
PAMPs: Pathogen-associated molecular patterns
PD-1: Programmed cell death protein 1
PD-L1: Programmed death-ligand 1
PD-L1B: Programmed death-ligand 1B
PDGF: Platelet-derived growth factor
Pea3: Polyomavirus enhancer activator 3
PKR: Protein kinase R
PTEN: Phosphatase and tensin homolog
Rb: Retinoblastoma protein
Reo: Reovirus
RGD: Arginine-Glycine-Aspartic Acid
RP2D: Recommended phase II dose
RR: Ribonucleotide reductase
SA: Sialic acid
SBRT: Stereotactic body radiation therapy
SCLC: Small-cell lung cancer
scFv: Single-chain variable fragment
Smac: Second mitochondria-derived activator of caspases
STING: Stimulator of interferon genes
SVA: Senecavirus A
SVV: Seneca valley virus
TAAs: Tumor-associated antigens
TAMs: Tumor-associated macrophages
TEM8: Tumor endothelial marker 8
TGF-β: Transforming growth factor-beta
TILs: Tumor-infiltrating lymphocytes
TK: Thymidine kinase
TLRs: Toll-like receptors
TME: Tumor microenvironment
TNBC: Triple-negative breast cancer
TNF-α: Tumor necrosis factor-alpha
TRAIL: Tumor necrosis factor-related apoptosis-inducing ligand
UL141: Human cytomegalovirus UL141 gene
US11: Herpes simplex virus US11 gene
US12: Herpes simplex virus US12 gene
VACV: Vaccinia virus
VEGF: Vascular endothelial growth factor
VSV: Vesicular stomatitis virus
WDSV: Walleye dermal sarcoma virus
