# Supplementary material for: Cracking the shield: oncolytic viruses versus the tumor-immune fortress
Source: Cancer Cell Int. 2026 May 25;26:197. doi: 10.1186/s12935-026-04331-1 (PMC13200443; doi:10.1186/s12935-026-04331-1)
Supplement: Supplementary file 1 — Additional file 1. [file 12935_2026_4331_MOESM1_ESM.docx]

Table S1: Examples of Genetically Modified Oncolytic Viruses

| **no.** | **Virus modified from** | **genome** | **Engineered virus name** | **Virus modification** | **Cancer type** | **Engineering approach** | **References** |
| --- | --- | --- | --- | --- | --- | --- | --- |
| 1. | NDV | ssRNA | NDV-MIP3α | Insertion of MIP-3α | colorectal carcinoma,  melanoma | Dendritic cell recruitment to enhance antitumor immunity | [1] (Huang et al., 2020) |
|  |  |  | rNDV-cIFNγ | Insertion of cIFNγ | malignant oral melanoma in humans and canines | Induction of cellular immunity via interferon expression | [2]  (Numpadit et al., 2023) |
|  |  |  | MEDI5395 | Insertion of granulocyte-macrophage colony stimulating factor (GM-CSF) | Melanoma, colon carcinoma | Enhance monocyte activation | [3]  (Harper et al., 2021) |
|  |  |  | rNDV-PTEN | Insertion of PTEN | Glioblastoma | Inhibits AKT/mTOR signaling to induce apoptosis | [4](Kim et al., 2024) |
|  |  |  | MEDI9253 | Insertion of IL-12 | Colon cancer | Activates NK and cytotoxic T cells | [5] (Najmuddin et al., 2020) |
|  |  |  | NDV(F3aa)-GFP | Three-amino-acid substitution in F protein | Head and neck squamous cell carcinoma (HNSCC) | Enhances oncolysis and tumor regression | [6] (Li et al., 2010) |
|  |  |  | rLaSota V.F. virus | Modified F protein cleavage site | Various cancer | Enhances IFN sensitivity and safety. | [7]  (Elankumaran et al., 2010) |
|  |  |  | rBC-Edit virus | Mutated V protein | various types of cancer | Promotes IFN production, restricts replication in normal cells | [7]  (Elankumaran et al., 2010) |
|  |  |  | rFMW/GFP | GFP-labeled fragment insertion | Anaplastic thyroid cancer | Reporter virus for infection tracking | [8](Jiang et al., 2018) |
|  |  |  | rZJ1-VS | V-deficient genotype VII  recombinant NDV | Various cancers | Lost the ability to reduce phospho-STAT1 and induced higher  expression of IFN-responsive genes in infected cells. | [18](Qiu et al., 2016) |
| 2. | Reovirus | dsRNA | rsT3D-L | Insertion of RGD peptide | Various cancers | Enhances cell tropism via integrin targeting | [9] (Kawagishi et al., 2020) |
|  |  |  | Super Virus 5 | Site-directed mutagenesis | Various cancers | Improves infectivity in cancer cells | [10] (Cristi et al., 2023) |
|  |  |  | rS1-GM-CSF | Insertion of murine /human GM-CSF | Pancreatic cancer | Stimulates dendritic and T cell activation | [11] (Kemp et al., 2018) |
| 3. | VSV | ssRNA | rVSV-UL141 | Insertion of HCMV UL141gene | Multifocal HCC | Downregulates CD155 to escape NK-mediated clearance | [12] (Altomonte et al., 2008) |
|  |  |  | VSV-IFNβ-NIS | Insertion of IFNβ and NIS | Various cancers | Enhances selectivity and allows imaging | [13] (Velazquez-Salinas et al., 2017) |
|  |  |  | rVSV-mIL12-mGM-CSF | Fusion of IL-12 and GM-CSF | Glioblastoma | Activates innate/adaptive immunity via systemic delivery | [14] (Ryapolova et al., 2023) |
|  |  |  | VSV-Δ51M-hIL-12 | Δ51M deletion + IL-12 insertion | Various cancers | Reduces M protein toxicity; boosts immune infiltration | [15] (Abdulal et al., 2023) |
|  |  |  | VSV-FH | Substitution with measles F/H proteins | Various cancers | Improved neurotolerance and cancer-cell killing | [16] (Nagalo et al., 2020) |
|  |  |  | VSV-12′GFP | Insertion of two reporter genes, shifting the NPMGL genes | Various cancers | High cancer-specific infection; attenuated in normal cells | [17] (Van Den Pol & Davis, 2012) |
| 4. | HSV | dsDNA | T-vec | Deletion of RL and US12 | Melanoma | Productive infection in malignant but not normal cells | [19] (Pol et al., 2015) |
|  |  |  | DELYTACT | Deletions in α34.5, α47, ICP6 | Recurrent glioblastoma | Enhances NK and CD8+ T cell activation | [20] (Todo et al., 2022) |
|  |  |  | oHSV1-IL15B | Insertion of IL-15/IL-15Rα complex | Colon cancer | Stabilizes IL-15 improving the trans-presentation to NK cells and CD8+ T cells | [21] (Hu et al., 2024) |
|  |  |  | oHSV1-aPD1 | Insertion of anti-PD-1 antibody | Colon cancer | Checkpoint blockade with cytokine synergy | [21] (Hu et al., 2024) |
|  |  |  | G161 | Insertion of IL-12, IL-15, PD-L1B | Breast cancer | Induces T, NK, and myeloid immune infiltration | [22] (Deng et al., 2023) |
|  |  |  | M032 | Insertion of IL12 | Glioblastoma | Promotes anti-angiogenic and immune response | [23] (Patel et al., 2016) |
|  |  |  | R5111 | Heparan sulfate site ablation + IL-13 insertion | Glioma | Improves tumor targeting, reduces off-target binding | [24](Zhou et al., 2002) |
|  |  |  | M002 | γ134.5-deleted HSV + IL-12 | Glioblastoma | Virus is selective and more effective in glioma cell killing | [25] (Wollmann  et al., 2012) |
|  |  |  | G207 | γ134.5 deletion + lacZ insertion | Gliomas | Safe replication in tumor cells only | [26] (Friedman et al., 2021) |
|  |  |  | RP2 (Replimune) | Insertion of  GM-CSF | Multiple cancers | Replication-competent, cytokine-enhanced HSV-1 | [27] (Harrington et al., 2022) |
|  |  |  | rQNestin34.5 | ICP34.5 under nestin promoter | Glioma | Enhanced replication and cytotoxicity in glioma | [28] (Kambara et al., 2005) |
|  |  |  | ICP6 mutant | ICP6/γ134.5 mutation | Glioma | Increases selectivity | [29] (Aghi et al., 2008) |
|  |  |  | MGH-1 | Deletion of ribonucleotide reductase (RR) and the neurovirulence factor γ34.5 | Gliosarcoma | Through deletion of RR and γ34.5, a weaker virus is generated for safety measures. | [30](Kramm et al., 1997) |
| 5. | Adenovirus | dsDNA | NG-350A | Insertion of anti CD40 antibody | Various Cancers | Blood stable, selective and Potent to advanced and metastatic cancer. | [31] (Naing et al., 2024) |
|  |  |  | NG-641 | Insertion of fibroblast activation protein-directed bi-specific T-cell activator antibody (FAP-TAc) and CXCL9/CXCL10/IFNα | Advanced or metastatic epithelial tumors | FAP-Tac targets immunosuppressive cancer-associated fibroblasts, while the CXCL9/CXCL10/IFNα activate the immune cells and recruit them. | [32] (Simon et al., 2022) |
|  |  |  | Ad5sPVR | Insertion of the soluble extracellular domain of poliovirus receptor (sPVR) | Various cancers | Overcomes the suppressive effects of checkpoints and insufficient costimulatory signals | [33]  (Zhang et al., 2020) |
|  |  |  | Ad-E6E7 | Insertion of inactive  mutant E6 an E7 human papillomavirus (HPV) transforming proteins | HPV associated cancers | Upon expression of E6 and E7 proteins, the host immune system is signaled to mount a cytotoxic T-lymphocyte (CTL) response against tumor cells expressing HPV E6 and E7. | [34] (*NCI Drug Dictionary*, n.d.) |
|  |  |  | ORCA-010 | E1AΔ24 deletion, T1 mutation, and RGD modification | Various cancers | The E1AΔ24 deletion is safety enhancing, the T1 mutation makes the virus more potent against tumor cells while the RGD modification is to increase infectivity. | [35] (Dong et al., 2014) |
|  |  |  | LOAd703 | Insertion of TMZ-CD40L and 4-1BBL | advanced pancreatic ductal adenocarcinoma. | To selectively lyse cancer cells, activate cytotoxic T cells and induce tumor regression. | [36]  (Musher et al., 2024) |
|  |  |  | ONCOS-102 (Targovax) | Insertion of granulocyte-macrophage colony stimulating factor (GM-CSF) | Various advanced cancers | Immune stimulation | [37]  (*ClinicalTrials.gov*, n.d.) |
|  |  |  | TILT-123 | Insertion of IL-2 and TNFα | Various advanced solid tumors | Stimulate, propagate and recruit T-cells, leading to tumor microenvironment re-invigoration. | [38] (Santos et al., 2023) |
|  |  |  | Ad5 [E1-, E2b-]-CEA | deletions in the E1, E2b and insertion of carcinoembryonic antigen (CEA) | Various cancers | Induces cell-mediated immunity which result in tumor growth inhibition despite the presence of pre-existing Ad5 immunity | [39] (Gabitzsch et al., 2010) |
|  |  |  | NSC-CRAd-S-pk7 | Insertion of Survivin promoter (S) and  fiber protein polylysine modification (pk7) loaded onto Neural stem cells (NSCs) | glioma | The virus is modified to present anti-neoplastic activity while bing loaded onto the NSCs, which preferentially migrate towards tumors. | [40]  (Mooney et al., 2018) |
|  |  |  | AdAPT-001 | Deletion of 50 targeted base pairs of two Pea3 transcription factor sites and one E2F1 transcription factor site. Insertion of a TGF-ß Trap. | Solid tumors | Selectivity enhancement . | [51]  (Conley et al., 2023) |
|  |  |  | CG0070 | Insertion of human granulocyte macrophage-colony stimulating factor (GM-CSF). E1A protein expression driven by human E2F-1 promoter. | Bladder cancer | It preferentially replicates in Rb protein-defective cells, typical of bladder cancer. As a result the GM-CSF produced activates the host immune response. | [52]  (Potts et al., 2012) |
|  |  |  | Ad-CALR/MAGE-A3 | Insertion of CALR and MAGE-A3 | non-small cell lung cancer cells | Enhances classical DC activation, induce CTLs. | [53]  (Liu et al., 2012) |
| 6. | Myxoma virus (MYXV) | dsDNA | vPD1/IL12 | Insertion of soluble PD1 inhibitor and IL12 | triple negative breast cancer | The systemic function of vPD1/IL12 is of high efficacy despite the low infection rates of the virus. | [41] (Thomas et al., 2023) |
|  |  |  | MC509-N1 | Insertion of TG1 and TG2 | Various cancers | TG1 should modify the microenvironment immune state, and TG2 allows the virus to evade the host immune defense. | [42] (Gansukh et al., 2021) |
|  |  |  | MyxV_CD47/IFN | Insertion of CD47 and IFN-γ | Various cancers | The overexpression of CD47 limits the rapid elimination of infected cells, IFN-γ enhances anticancer immunity induced | [43] (Woo et al., 2023) |
|  |  |  | vMyx-IL-15 | Insertion of IL-15 | Melanoma | Promotes infiltration of neutrophils which causes inflammation of the tumor bed. | [44] (Rahman & McFadden, 2020) |
|  |  |  | MYXVorfC | Insertion of orfC gene of walleye dermal sarcoma virus (WDSV) | Various types of cancer | Induces apoptosis | [45] (Ashton et al., 2020) |
|  |  |  | MYXVΔserp2 | Deletion of serp2 | Various types of cancer | Serp2 is an anti-apoptotic and a virulence factor. This deletion enhances oncolytic effects and lessens the viral pathogenesis. | [46] (MacNeill et al., 2018) |
| 7. | Senecavirus A (SVA) | ssRNA | SVA-CH-01-2015 | Insertion of p16^INK4A^ | Various types of cancer | p16^INK4A^ is a cell cycle-dependent protein kinase inhibitor 2A (CDKN2A), it will regulate the cell replication cycle and suppress tumor growth | [47] (Gong et al., 2023) |
|  |  |  | SVV-37 (SVV-CXCL9) | Insertion of CXCL9 | Various types of cancer | CXCL9, known to mediate the recruitment of activated CD8+ cytotoxic T cells and CD4+ helper T cells | [48] (Zhao et al., 2024) |
| 8. | Parvovirus | ssDNA | H-1RGD | Insertion of the RGD-4C next to a nitro-carboxy group of Ala 441 of the VP2 sequence | Various Cancers | These residues prevented the entry of H-1PV into cells usually permissive for wild-type virus infection. | [49] (Allaume et al., 2012) |
| 9. | VacciniaVirus (VACV) | dsDNA | JX-594 (Jennerex Biotherapeutics Green Cross Corporation) | Inactivation thymidine kinase (TK) insertion of granulocyte-macrophage colony-stimulating factor (GM-CSF) | Various Cancers | Targets cancer cells through replication dependent cell lysis, and stimulation of antitumor immunity | [50] (Parato et al., 2011) |
|  |  |  | TG6050 | Insertion of single-chain human interleukin-12 (hIL-12) and full-length anti-cytotoxic T-lymphocyte-associated antigen-4 (CTLA-4) monoclonal antibody. | Metastatic non-small cell lung cancer | Activates inflammatory state in cold tumors | [54]  (Nakao et al., 2020) |
|  |  |  | vvDD-IL15-ra | superagoinst IL-15, a fusion protein of IL-15 and IL-15Ralpha. | Ovarian and colon | Results in significantly more regression of the disease | [55](Kowalsky et al., 2018) |
|  |  |  | SJ-600 | Insertion of CD55 protein | Various types of cancel | Evades neutralization; this elongates antitumor efficacy | [56] (Lee et al., 2023) |
