# Supplementary material for: Cracking the shield: oncolytic viruses versus the tumor-immune fortress
Source: Cancer Cell Int. 2026 May 25;26:197. doi: 10.1186/s12935-026-04331-1 (PMC13200443; doi:10.1186/s12935-026-04331-1)
Supplement: Supplementary file 2 — Additional file 2. [file 12935_2026_4331_MOESM2_ESM.docx]

**Table S2: Ongoing and Concluded Clinical Studies Assessing Oncolytic Virus Therapeutic Potential in Male and Female Patients**

| **Study Title** | **Conditions** | **Interventions** | **Age** | **Phases** | **Cases** | **Published Result** | **Virus Type** | Study Design | **Findings/Status** | **Clinical Trials ID** |
| --- | --- | --- | --- | --- | --- | --- | --- | --- | --- | --- |
| A Clinical Study on Oncolytic Virus Injection (R130 OV) for the Treatment of Advanced Solid Tumors | Advanced Solid Tumors | Biological: Recombinant oncolytic herpes simplex virus type 1 (R130)   - R130, a modified herpes simplex virus-Ⅰ (HSV-1) containing the gene coding for anti-CD3 scFv/CD86/PD1/HSV2-US11 | 18 - 75 | Early Phase 1 | 20 | No | Recombinant oncolytic herpes simplex virus type 1 (R130) | Single-armed clinical trial to evaluate the safety and efficacy of the recombinant herpes simplex virus Ⅰ, R130 in patients with advanced solid tumors | Not published | NCT05961111 |
| A Clinical Study on Oncolytic Virus Injection (R130) for the Treatment of Advanced Solid Tumors | Different types of cancers (Sarcoma -Breast - Pancreatic - Colorectal - Gastric - Liver - Lung -Gynecologic ) | Drug: Recombinant oncolytic herpes simplex virus type 1 (R130) | 18 - 75 | Early Phase 1 | 20 | No | Recombinant oncolytic herpes simplex virus type 1 (R130) | A Clinical Safety and Efficacy Study on Oncolytic Virus Injection (R130) for the Treatment of Advanced Solid Tumors | Not published | NCT05860374 |
| A Clinical Study on Oncolytic Virus Injection (R130 OV) for the Treatment of Relapsed/Refractory Head and Neck Cancer | Advanced Solid Tumors | Drug: Recombinant oncolytic herpes simplex virus type 1 (R130) | 18 - 75 | Early Phase 1 | 9 | No | Recombinant oncolytic herpes simplex virus type 1 (R130) | single-armed clinical trial to evaluate the safety and efficacy of the recombinant herpes simplex virus Ⅰ, R130 in patients with relapsed/refractory head and neck cancer | Not published | NCT05830240 |
| Oncolytic Virus Plus Anti-PD1 and Chemotherapy as Preoperative Therapy for Patients With BRPC/LAPC | BRPC/?LAPC | Drug: Oncolytic virus Plus Anti-PD1 and Chemotherapy | 18 – 75 | Phase 1 | 20 | No | Oncolytic virus Plus Anti-PD1 and Chemotherapy | single arm and prospective study is to explore the safety and efficacy of Oncolytic virus Plus Anti-PD1 and Chemotherapy as Preoperative therapy for Patients with Borderline Resectable and Locally Advanced Pancreatic Cancer | Not published | NCT06346808 |
| A Phase I Clinical Study of Intratumoral Injection Oncolytic Vaccinia Virus GC001 in Patient With Advanced Solid Tumors | Advanced Solid Tumors | Drug: Oncolytic virus Plus Anti-PD1 and Chemotherapy | 18 - 75 | Phase 1 | 20 | No | Oncolytic Vaccinia Virus GC001 | single-arm phase I clinical study aimed at assessing the safety, tolerability, viral distribution and shedding patterns, pharmacodynamics, immunogenicity, and antitumor efficacy of GC001 oncolytic virus injection in patients with advanced solid tumors following a single administration. | Not published | NCT06508307 |
| Intravenous Injection of Oncolytic Virus Injection (RT-01) in Patients With Relapsed or Refractory T-cell Lymphoma | Relapsed or Refractory T-cell Lymphoma | Biological: Oncolytic Virus Injection(RT-01) | 18 | Phase 1 | 6 | No | Oncolytic Virus Injection(RT-01) | A Sing-Arm, Open Clinical Pharmacology Study of Intravenous Injection of Oncolytic Virus Injection (RT-01) in Patients With Relapsed or Refractory T-cell Lymphoma | Not published | NCT05387226 |
| A Study of CodaLytic, an Intratumoral Oncolytic Virus, in Patients With Breast Cancer | Breast Neoplasms Neoplasm Metastasis | Biological: CodaLytic | 18 | Phase 1 | 0  (trial was withdrawn before enrolling any subjects) | No | CodaLytic | Phase 1 Study of CodaLytic, an Intratumoral Influenza-based Oncolytic Virus, in Patients With Metastatic or Otherwise Inoperable Breast Cancer | Not published | NCT05600582 |
| The Application of Novel Oncolytic Virus in Late Stage Solid Tumors | Malignant Tumor | Drug: Oncolytic Virus SDJ001 Drug: Oncolytic Virus YD06-1 | 18 | Phase 1 | 24 | Yes | Oncolytic Virus SDJ001  Oncolytic Virus YD06-1 | Evaluate the efficacy and safety of novel oncolytic virus in late stage solid tumors. | Published: The purpose of this study is to evaluate the efficacy and safety of novel oncolytic virus in late stage solid tumors. | NCT06080984 |
| Oncolytic Virus (OVV-01) Injection in the Treatment of Patients With Advanced Solid Tumors | Neoplasms | Drug: oncolytic virus (OVV-01) injection | 18 - 70 | Phase 1 | 50 | No | oncolytic virus (OVV-01) injection | A Single-arm, Open-label Clinical Study to Evaluate the Safety, Tolerability and Preliminary Efficacy of Oncolytic Virus (OVV-01) Injection Combined With or Without Immune Checkpoint Inhibitors in the Treatment of Patients With Advanced Solid Tumors | Published: Phase Ia: To investigate the safety, tolerability and efficacy of OVV-01 injection in the treatment of patients with advanced solid tumors (OVV-01 single dose gradient exploration).Phase Ib: To evaluate the safety, tolerability and efficacy of OVV-01 injection combined with immune checkpoint inhibitors pembrolizumab (anti-PD-1 monoclonal antibody) or atezolizumab (anti-PD-L1 monoclonal antibody) in the treatment of patients with advanced solid tumors (OVV-01 combined with PD-1/PD-L1 monoclonal antibody dose gradient exploration);Phase Ic: A cohort expansion of Phase Ib to further analyze the efficacy and safety of OVV-01 injection combined with immune checkpoint inhibitor injection in the treatment of advanced solid tumors. | NCT04787003 |
| A Phase I Clinical Study of Intratumoral Injection Oncolytic Vaccinia Virus GC001 in Patient With Advanced Solid Tumors | Sarcoma Cervical Cancer Colon Cancer Lung Cancer Ovarian Cancer Pancreatic Cancer Hepatocellular Carcinoma Breast Cancer Gastric Cancer | Biological: A Phase I Clinical Study of Intratumoral Injection Oncolytic Vaccinia Virus GC001 in Patients With Advanced Solid Tumors | 18 - 75 | Phase 1 | 21 | No | A Phase I Clinical Study of Intratumoral Injection Oncolytic Vaccinia Virus GC001 in Patients With Advanced Solid Tumors | A Phase I Study Evaluating the Safety, Tolerability, Biodistribution and Shedding of the Virus, Pharmacodynamics, Immunogenicity, and Antitumor Activity of GC001 Oncolytic Vaccinia Virus Injection in Patient With Advanced Solid Tumors. | Not published | NCT06508307 |
| Safety of Recombinant Human IL-21-expressing Oncolytic Vaccinia Virus Injection (hV01) in Advanced Tumors | Advanced Solid Tumors | Biological: Recombinant human IL-21-expressing oncolytic vaccinia virus injection | 18 - 75 | Phase 1 | 24 | No | Recombinant human IL-21-expressing oncolytic vaccinia virus injection | A Phase I Dose Escalation Study to Evaluate the Safety, Tolerance, Pharmacokinetics, and Biological Properties of Recombinant Human IL-21-expressing Oncolytic Vaccinia Virus Injection (hV01) in Patients With Advanced Malignant Solid Tumors | Not published | NCT05914376 |
| A Study of Recombinant Oncolytic Virus M1(VRT106) in Patients with Solid Tumors | Solid Tumor | Biological: VRT106 | 18 - 75 | Phase 1 | 30 | Yes | VRT106 | A Phase I Clinical Study to Evaluate the Safety, Tolerability, Biodistribution Characteristics, Biological Effects and Initial Efficacy of Recombinant Oncolytic Virus M1 for Injection (VRT106) in the Treatment of Patients With Locally Advanced or Metastatic Solid Tumors | Not published | NCT06368921 |
| TNF? and IL-2 Coding Oncolytic Adenovirus TILT-123 Monotherapy (TUNIMO) | Solid Tumor | Biological: TILT-123 | 18 | Phase 1 | 18 | No | TILT-123 | A Phase 1, Open-Label, Dose-escalation Clinical Trial of Tumor Necrosis Factor Alpha and Interleukin-2 Coding Oncolytic Adenovirus (TILT-123) in Patients With Injectable Solid Tumors | Published: This is an open-label, phase 1, dose-escalation, multicenter trial evaluating the safety of oncolytic adenovirus TILT-123 as monotherapy in advanced solid tumor patients. | NCT04695327 |
| A Study of an Intratumoral Oncolytic Virus in Patients With Advanced Metastatic Solid Tumors | Metastatic Cancer Solid Tumors Advanced Cancer | Biological: ASP9801 Combination Product: Pembrolizumab | 18 | Phase 1 | 72 | No | ASP9801 Pembrolizumab | A Phase 1, Open-label Study of ASP9801, an Oncolytic Virus, Administered by Intratumoral Injection as a Single Agent and in Combination With Pembrolizumab in Subjects With Advanced/Metastatic Solid Tumors | Not published | NCT03954067 |
| Oncolytic Virus Plus Anti-PD1 and Chemotherapy as Preoperative Therapy for Patients With BRPC/?LAPC | Pancreatic Cancer | Drug: Oncolytic virus Plus Anti-PD1 and Chemotherapy | 18 - 75 | Phase 1 | 20 | No | Oncolytic virus Plus Anti-PD1 and Chemotherapy | Oncolytic Virus Plus Anti-PD1 and Chemotherapy as Preoperative Therapy for Patients With Borderline Resectable and Locally Advanced Pancreatic Cancer | Not published | NCT06346808 |
| Safety and Efficacy of the ONCOlytic VIRus Armed for Local Chemotherapy, TG6002/5-FC, in Recurrent Glioblastoma Patients (ONCOVIRAC) | Glioblastoma Brain Cancer | Drug: Combination of TG6002 and 5-flucytosine (5-FC, Ancotil®) | 18 | Phase 1 and 2 | 78 | No | Combination of TG6002 and 5-flucytosine (5-FC, Ancotil®) | Safety and Efficacy of the ONCOlytic VIRus Armed for Local Chemotherapy, TG6002/5-FC, in Recurrent Glioblastoma Patients | Not published | NCT03294486 |
| OH2 Oncolytic Viral Therapy in Non-Muscle-Invasive Bladder Cancer | Non-muscle-invasive Bladder Cancer | Biological: OH2 injection | 18 - 80 | Phase 1 and 2 | 30 | No | OH2 injection | Oncolytic Virus (OH2) Adjuvant Therapy After Transurethral Resection of Bladder Tumor in Non-Muscle-Invasive Bladder Cancer Who Have Failed First-line Prophylactic Intravesical Instillation Therapy: a Phase Ⅰb/Ⅱ Clinical Trial | Not published | NCT05232136 |
| OH2 Oncolytic Viral Therapy in Solid Tumors | Solid Tumor Gastrointestinal Cancer | Biological: OH2 injection, with or without irinotecan or HX008 | 18 - 75 | Phase 1 and 2 | 300 | No | OH2 injection, with or without irinotecan or HX008 | Phase I/II Study of OH2 Injection, an Oncolytic Type 2 Herpes Simplex Virus Expressing Granulocyte Macrophage Colony-Stimulating Factor, in Malignant Solid Tumors | Published: This phase I/II study evaluates the safety and efficacy of OH2 as single agent or in combination with HX008, an anti-PD-1 antibody, in patients with malignant solid tumors (gastrointestinal cancers, head and neck cancers, soft tissue sarcomas).OH2 is an oncolytic virus developed upon genetic modifications of the herpes simplex virus type 2 strain HG52, allowing the virus to selectively replicate in tumors. Meanwhile, the delivery of the gene encoding human granulocyte macrophage colony-stimulating factor (GM-CSF) may induce a more potent antitumor immune response | NCT03866525 |
| LOAd703 Oncolytic Virus Therapy for Pancreatic Cancer | Pancreatic Cancer | Genetic: delolimogene mupadenorepvec Drug: gemcitabine Drug: nab-paclitaxel Biological: atezolizumab | 18 | Phase 1 and 2 | 51 | No | LOAd703, an Armed Oncolytic Adenovirus for Pancreatic Cancer | this study is to see if LOAd703 (an oncolytic adenovirus) can be safely given to patients with pancreatic cancer. The study will also evaluate whether or not intratumoral injection of LOAd703 will support current standard of care treatment to reduce the size of the tumor and improve survival of the patients. | Published: The purpose of this study is to see if LOAd703 (an oncolytic adenovirus) can be safely given to patients with pancreatic cancer. The study will also evaluate whether or not intratumoral injection of LOAd703 will support current standard of care treatment to reduce the size of the tumor and improve survival of the patients. | NCT02705196 |
| OH2 Oncolytic Viral Therapy in Central Nervous System Tumors | Central Nervous System Tumors | Biological: OH2 injection | 18 | Phase 1 and 2 | 28 | No | OH2 injection | A Clinical Study of Oncolytic Virus (OH2) Injection in the Treatment of Patients Undergoing Surgery After Recurrence of Central Nervous System Tumors | Published: In the first phase, it mainly explores the safety, tolerability and preliminary effectiveness of two doses of OH2 injection in the treatment of patients with recurrent central nervous system tumors; to evaluate the biodistribution and virus shedding of OH2 injection administered in the tumor cavity; to evaluate the level of anti-HSV2 antibody in patients when OH2 injection is administered intracavitary to tumor; to determine the phase II recommended dose (RP2D) of OH2 injection in the treatment of recurrent glioblastoma.  Phase IIa, to evaluate the preliminary efficacy of OH2 injection in the treatment of patients with recurrent glioblastoma after surgery, and to further evaluate the safety of OH2 in the treatment of relapsed glioblastoma. | NCT05235074 |
| Intraperitoneal Injection of Oncolytic Viruses H101 for Patients With Refractory Malignant Ascites | Refractory Malignant Ascites | Drug: Oncorine (H101) | 18 | Phase 2 | 25 | Yes | Oncorine (H101) | A Phase II Study of Intraperitoneal Injection of Oncolytic Viruses H101 for Patients With Refractory Malignant Ascites | Not published | NCT04771676 |
| Intra-tumor Injection of Oncolytic Viruses H101 Combined With or Without Radiotherapy in Refractory/?Recurrent Gynecological Malignancies | Genital Neoplasms, Female | Drug: H101 | 18 | Phase 2 | 120 | No | H101 | Evaluate the effectiveness and safety of oncolytic viruses H101 intra-tumor injection combined with or without radiotherapy in refractory or recurrent gynecological malignancies. Further research the mechanism of oncolytic viruses H101. | Not published | NCT05051696 |
| Oncolytic Virus Plus PD-1 Inhibitor to Patients With Advanced Pancreatic Cancer (PTCA199-8) | Pancreatic Cancer | Drug: H101 Drug: Camrelizumab | 18 - 80 | Phase 2 | 30 | No | H101  Camrelizumab | Evaluate the efficacy of oncolytic virus plus PD-1 inhibitor to Patients with Advanced Pancreatic Cancer. | Published: The purpose of this study is to evaluate the efficacy of oncolytic virus plus PD-1 inhibitor to Patients with Advanced Pancreatic Cancer. | NCT06196671 |
| OH2 Oncolytic Viral Therapy in Advanced Bladder Cancer | Advanced Bladder Carcinoma | Biological: OH2 injection | 18 - 75 | Phase 2 | 45 | No | OH2 injection | Efficacy and Safety Study of Oncolytic Virus (OH2) Intratumoral Injection in Locally Advanced or Metastatic Bladder Cancer a Phase Ⅱ Clinical Trial | Not published | NCT05248789 |
| SBRT and Oncolytic Virus Therapy Before Pembrolizumab for Metastatic TNBC and NSCLC (STOMP) | Metastatic Non-small Cell Lung Cancer Metastatic Triple-negative Breast Cancer | Biological: ADV/HSV-tk Drug: Valacyclovir Radiation: SBRT Drug: Pembrolizumab | 18 | Phase 2 | 57 | Yes | ADV/HSV-tk | Phase II Window of Opportunity Trial of Stereotactic Body Radiation Therapy and In Situ Oncolytic Virus Therapy in Metastatic Triple Negative Breast Cancer and Metastatic Non-Small Cell Lung Cancer Followed by Pembrolizumab | Not published | NCT03004183 |
| Evaluate Efficacy, Immunological Response of Intratumoral/Intralesional Oncolytic Virus (OBP-301) in Metastatic Melanoma | Melanoma Stage III Melanoma Stage Iv | Drug: OBP-301 | 18 | Phase 2 | 4 | No | OBP-301 | Open-label, Multi-center Phase IIa Study to Evaluate the Efficacy, Safety, and Immunological Response of OBP-301, Telomerase Specific Replication-competent Oncolytic Adenovirus in Patients With Unresectable Metastatic Melanoma | Not published | NCT03190824 |
| Safety Study of GL-ONC1, an Oncolytic Virus, in Patients With Advanced Solid Tumors | Advanced Cancers (Solid Tumors) | Biological: GL-ONC1 | 18 | Phase 1 | 43 | No | GL-ONC1 | Phase I Study of the Safety, Tolerability,and Tumor-Specific Replication of the Intravenous Administration of Green Fluorescent Protein Encoded Genetically Engineered Attenuated Vaccinia Virus, GL-ONC1, in Patients With Advanced Solid Organ Cancers. | Not published | NCT00794131 |
| A Clinical Study on Oncolytic Virus Injection (R130) for the Treatment of Relapsed/Refractory Ovarian Cancer | Ovarian Cancer Peritoneal Carcinomatosis Fallopian Tube Cancer | Drug: Recombinant oncolytic herpes simplex virus type 1 (R130) | 18 - 75 | Early Phase 1 | 10 | No | Recombinant oncolytic herpes simplex virus type 1 (R130) | A Clinical Safety and Efficacy Study on Oncolytic Virus Injection (R130) for the Treatment of Relapsed/Refractory Ovarian Cancer | Not published | NCT05801783 |
| A Study of Intratumoral Administration of Oncolytic Virus Injection (RT-01) in Patients With Advanced Solid Tumors | Advanced Solid Tumor | Biological: Oncolytic Virus Injection(RT-01) | 18 | Phase 1 | 7 | No | Oncolytic Virus Injection(RT-01) | A Single-Arm, Open-Label, Dose Escalation Study to Evaluate Safety and Efficacy of Intratumoral Injection of Oncolytic Virus Injection (RT-01) in Patients With Advanced Solid Tumors | Not published | NCT05205408 |
| A Study of GL-ONC1, an Oncolytic Vaccinia Virus, in Patients With Advanced Peritoneal Carcinomatosis | Peritoneal Carcinomatosis | Biological: GL-ONC1 | 18 | Phase 1 and 2 | 9 | No | GL-ONC1 | Phase I/II Study of Intraperitoneal Administration of GL-ONC1, a Genetically Modified Vaccinia Virus, in Patients With Peritoneal Carcinomatosis | Published: The purpose of this study is to determine whether GL-ONC1, an attenuated vaccinia virus, is safe when administered to patients with peritoneal carcinomatosis via an infusion within the abdominal cavity through an implanted catheter. The study seeks also to arrive at a recommended dose and schedule for future investigations, evidence of anti-tumor activity, detection of virus in body fluids, analysis of viral delivery to tumor and normal cells, and to evaluate if there is an antibody response to vaccinia virus. | NCT01443260 |
| A Study of Oncolytic Virus Injection (RT-01) in Patients With Extensive-Stage Small Cell Lung Cancer | Advanced Solid Tumor | Biological: Oncolytic Virus Injection(RT-01) | 18 | Phase 1 | 20 | No | Oncolytic Virus Injection (RT-01) | A Single-Arm, Open-Label, Exploratory Study to Evaluate Safety and Efficacy of Oncolytic Virus Injection (RT-01) in Patients With Extensive-Stage Small Cell Lung Cancer | Not published | NCT05205421 |
| A Clinical Study on Oncolytic Virus Injection (R130) for the Treatment of Relapsed/?Refractory Advanced Solid Tumors | Lung Cancer Bronchial Cancer Non Small Cell Lung Cancer Small Cell Lung Cancer Sarcoma Colorectal Cancer Gastric Cancer Liver Cancer Breast Cancer Pancreatic Cancer Head and Neck Cancer Ovarian Cancer | Drug: Recombinant oncolytic herpes simplex virus type 1 (R130) | 18 - 75 | Early Phase 1 | 24 | No | Recombinant oncolytic herpes simplex virus type 1 (R130) | A Clinical Safety and Efficacy Study on Oncolytic Virus Injection (R130) for the Treatment of Relapsed/Refractory Advanced Solid Tumors | Not published | NCT05070221 |
| Study of Oncolytic Virus in Combination With HX-008 and Axitinib in Melanoma Patients With Liver Metastasis | Melanoma Stage IV | Drug: Recombinant Oncolytic HSV-2 Therapeutic Injecta (Vero Cell) for Human Use (rHSV2hGM-CSF) Drug: Recombinant humanized anti-PD-1 monoclonal antibody for injection Drug: Axitinib | 18 - 75 | Phase 1 | 30 | No | Recombinant Oncolytic HSV-2 Therapeutic Injecta (Vero Cell) for Human Use (rHSV2hGM-CSF)  ;Recombinant humanized anti-PD-1 monoclonal antibody for injection  Axitinib | Safety and Efficacy Study of Oncolytic Virus（Intratumoral Injection）in Combination With HX-008（Intravenous Injection）and Axitinib in Melanoma Patients With Liver Metastasis Who Lack or Become Refractory to Standard Treatment | Not published | NCT05070221 |
| Study of Oncolytic Virus in Combination With HX-008 and Radiotherapy in Melanoma Patients With Liver Metastasis | Melanoma Stage IV | Drug: Recombinant Oncolytic HSV-2 Therapeutic Injecta (Vero Cell) for Human Use (rHSV2hGM-CSF) Drug: Recombinant humanized anti-PD-1 monoclonal antibody for injection Radiation: RT | 18 - 75 | Phase 1 | 15 | No | Recombinant Oncolytic HSV-2 Therapeutic Injecta (Vero Cell) for Human Use (rHSV2hGM-CSF)  Recombinant humanized anti-PD-1 monoclonal antibody for injection  RT | Safety and Efficacy Study of Oncolytic Virus（Intratumoral Injection）in Combination With HX-008（Intravenous Injection）and Radiotherapy for Liver Metastasis in Melanoma Patients With Liver Metastasis Who Lack or Become Refractory to Standard Treatment | Not published | NCT05068453 |
| A Clinical Study on Oncolytic Virus Injection (R130) for the Treatment of Advanced Bone and Soft Tissue Tumors | Osteosarcoma  Sarcoma Soft Tissue Sarcoma Bone Tumor | Drug: Recombinant oncolytic herpes simplex virus type (R130) | 16- 75 |  | 9 | No | Recombinant oncolytic herpes simplex virus type Ⅰ (R130) | A Clinical Safety and Efficacy Study on Oncolytic Virus Injection (R130) for the Treatment of Advanced Bone and Soft Tissue Tumors | Not published | NCT06171282 |
| Safety and Efficacy of CG0070 Oncolytic Virus Regimen for High Grade NMIBC After BCG Failure (BOND2) | Bladder Cancer | Biological: CG0070 | 18 | Phase 2 | 66 | Yes | CG0070 | An Open Label, Single Arm, Phase II, Multicenter Study of the Safety and Efficacy of CG0070 Oncolytic Vector Regimen in Patients With Non-Muscle Invasive Bladder Carcinoma Who Have Failed BCG (Bacillus Calmette-Guerin) Therapy and Refused Cystectomy | Not published | NCT02365818 |
| A Clinical Study on Oncolytic Virus Injection (R130) for the Treatment of Relapsed/Refractory Cervical and Endometrial Cancer | Cervical Cancer Endometrial Cancer Advanced Cancer | Drug: Recombinant oncolytic herpes simplex virus type ? (R130) | 18 - 75 | Early Phase 1 | 20 | No | Recombinant oncolytic herpes simplex virus type Ⅰ (R130) | A Clinical Safety and Efficacy Study on Oncolytic Virus Injection (R130) for the Treatment of Relapsed/Refractory Cervical and Endometrial Cancer | Not published | NCT05812677 |
| Exploratory Study of a Novel Oncolytic Vaccinia Virus RGV004 in the Treatment of Refractory/Relapsed B-cell Lymphoma | Relapsed or Refractory B-cell Lymphoma | Biological: RGV004 | 18 - 75 | Phase 1 | 25 | No | RGV004 | Exploratory Study of a Novel Oncolytic Vaccinia Virus Expressing Bispecific Antibody in the Treatment of Refractory/Relapsed B-cell Lymphoma | Not published | NCT04887025 |
| Virus DNX2401 and Temozolomide in Recurrent Glioblastoma (D24GBM) | Glioblastoma Multiforme Recurrent Tumor | Procedure: DNX2401 and Temozolomide | 18 - 75 |  | 31 | No | DNX2401 and Temozolomide | Phase I Trial of Combination of DNX-2401 (Formerly Named Delta-24-RGD) Oncolytic Adenovirus With a Short Course of Temozolomide for Treatment of Glioblastoma at First Recurrent | Published: Phase I trial, unicentric, uncontrolled. Intratumoral injection or intramural (into the resected tumor cavity) of DNX2401 into brain tissue will be followed by up to two 28 - day cycles of oral temozolomide (TMZ) in schedule of 7 days on/7 days off to evaluate safety of the combination. Completion of two full cycles of TMZ will be dependent upon tolerance and toxicity.The rationale in using the virus with chemotherapy begins with the lessons learned in many clinical trials in glioblastoma (GBM) about both the great difficulty of treating this disease with monotherapy and the limitations of the therapeutic virus. The best clinical results in recent years have been achieved with combinations of multiple therapeutics efforts, including, maximum resection and chemotherapy, immunotherapy and targeted therapies. There are very strong preclinical data about the synergy of DNX-2401 and TMZ proposed in our trial design. The dose-dense schemes of TMZ like the one we will use, have been developed with the aim to saturate o6-methylguanine-DNA-methyltransferase (MGMT). The published results to date have shown reasonable toxicity albeit with modest efficacy' these schemes are now in phase III trials. In addition, autophagy triggered by TMZ could help viral replication in the tumor cells 11.The last argument in favor of this virus + TMZ combination is the proved efficacy in killing GBM tumor stem cells. In vitro and animals models have shown this combination is much more effective that any of the treatments alone against GBM stem cells and the tumors derived from them. | NCT01956734 |
| A Clinical Study on Oncolytic Virus Injection (R130 OV) for the Treatment of Relapsed/Refractory Head and Neck Cancer | Head and Neck Cancer Esophageal Cancer Otorhinolaryngologic Neoplasms Ear Cancer Nose Cancer Laryngeal Cancer Pharyngeal Cancer | Drug: Recombinant oncolytic herpes simplex virus type 1 (R130) | 18 - 75 | Early Phase 1 | 9 | No | Recombinant oncolytic herpes simplex virus type 1 (R130) | A Clinical Safety and Efficacy Study on Oncolytic Virus Injection (R130) for the Treatment of Relapsed/Refractory Head and Neck Cancer | Not published | NCT05830240 |
| A Phase I/II Study of Pexa-Vec Oncolytic Virus in Combination With Immune Checkpoint Inhibition in Refractory Colorectal Cancer | Colorectal Cancer Colorectal Carcinoma Colorectal Adenocarcinoma Refractory Cancer Colorectal Neoplasms | Drug: Durvalumab Drug: Tremelimumab Biological: Pexa-Vec Biological: Pexa-Vec | 18 | Phase 1 and 2 | 34 | Yes | Durvalumab  Tremelimumab  Pexa-Vec | A Phase I/II Study of Pexa-Vec Oncolytic Virus in Combination With Immune Checkpoint Inhibition in Refractory Colorectal Cancer | Published: To determine the safety, tolerability and feasibility of Pexa-Vec oncolytic virus in combination with immune checkpoint inhibition in patients with refractory metastatic colorectal cancer. The proposed study is Phase I/II study of Pexa-Vec oncolytic virus at two dose levels in combination with immune checkpoint inhibition in patients with metastatic colorectal cancer. | NCT03206073 |
| A Study of Oncolytic Virus Injection (RT-01) in Combination With PD-1 Inhibitor in Patients With Advanced Solid Tumors | Advanced Solid Tumor | Biological: Oncolytic Virus Injection(RT-01) | 18 | Phase 1 | 50 | No | Oncolytic Virus Injection (RT-01) | A Single-Arm, Open-Label, Exploratory Study to Evaluate Safety and Efficacy of Oncolytic Virus Injection (RT-01) in Combination With PD-1 Inhibitor (Nivolumab) in Patients With Advanced Solid Tumors | Not published | NCT05228119 |
| A Clinical Study on Oncolytic Virus Injection (R130) for the Treatment of Relapsed/Refractory Bone and Soft Tissue Tumors | Osteosarcoma Sarcoma Sarcoma,Soft Tissue Bone Tumor | Drug: Recombinant oncolytic herpes simplex virus type ? (R130) | 14 - 75 | Early Phase 1 | 20 | No | Recombinant oncolytic herpes simplex virus type Ⅰ (R130) | A Clinical Safety and Efficacy Study on Oncolytic Virus Injection (R130) for the Treatment of Relapsed/Refractory Bone and Soft Tissue Tumors | Not published | NCT05851456 |
| Oncolytic Virus Ad-TD-nsIL12 for Primary Pediatric Diffuse Intrinsic Pontine Glioma | Oncolytic Virus Diffuse Intrinsic Pontine Glioma Adverse Drug Event | Biological: Ad-TD-nsIL12 | 1 _ 18 | Phase 1 | 18 | No | Ad-TD-nsIL12 | Oncolytic Virus Ad-TD-nsIL12 for Primary Pediatric Diffuse Intrinsic Pontine Glioma | Published: This is a drug safety assessment clinical trial with a 3+3 dose escalation design, to observe the safety, tolerability and toxicity of a novel oncolytic virus Ad-TD-nsIL12 intratumoral injection in primary DIPG patients (NCI-CTCAE V5.0). | NCT05717712 |
| Oncolytic Virus Ad-TD-nsIL12 for Progressive Pediatric Diffuse Intrinsic Pontine Glioma | Oncolytic Virus Diffuse Intrinsic Pontine Glioma Adverse Drug Event | Biological: Ad-TD-nsIL12 | 1 _ 18 | Phase 1 | 18 | No | Ad-TD-nsIL12 | Oncolytic Virus Ad-TD-nsIL12 for Progressive Pediatric Diffuse Intrinsic Pontine Glioma | Published: This is a drug safety assessment clinical trial with a 3+3 dose escalation design, to observe the safety, tolerability and toxicity of a novel oncolytic virus Ad-TD-nsIL12 intratumoral injection in primary DIPG patients (NCI-CTCAE V5.0). | NCT05717699 |
| LOAd703 Oncolytic Virus Therapy for Pancreatic Cancer | Pancreatic Cancer | Genetic: delolimogene mupadenorepvec Drug: gemcitabine Drug: nab-paclitaxel Biological: atezolizumab | 18 | Phase 1 and 2 | 51 | No | delolimogene mupadenorepvec  gemcitabine  nab-paclitaxel  atezolizumab | Phase I/IIa Trial Evaluating Safety of LOAd703, an Armed Oncolytic  Adenovirus for Pancreatic Cancer | Published: The purpose of this study is to see if LOAd703 (an oncolytic adenovirus) can be safely given to patients with pancreatic cancer. The study will also evaluate whether or not intratumoral injection of LOAd703 will support current standard of care treatment to reduce the size of the tumor and improve survival of the patients. | NCT02705196 |
| Intraperitoneal Injection of Oncolytic Viruses H101 for Patients With Refractory Malignant Ascites | Refractory Malignant Ascites | Drug: Oncorine (H101) | 18 | Phase 2 | 25 | No | Oncorine (H101) | A Phase II Study of Intraperitoneal Injection of Oncolytic Viruses H101 for Patients With Refractory Malignant Ascites | Not published | NCT04771676 |
| The Application of Novel Oncolytic Virus in Late Stage Solid Tumors | Malignant Tumor | Drug: Oncolytic Virus SDJ001 Drug: Oncolytic Virus YD06-1 | 18 | Phase 1 | 24 | No | Oncolytic Virus SDJ001  Oncolytic Virus YD06-1 | The Application of Novel Oncolytic Virus in Late Stage Solid Tumors | Published: The purpose of this study is to evaluate the efficacy and safety of novel oncolytic virus in late stage solid tumors. | NCT06080984 |
| Intra-tumor Injection of Oncolytic Viruses H101 Combined With or Without Radiotherapy in Refractory/Recurrent Gynecological Malignancies | Genital Neoplasms, Female | Drug: H101 | 18 | Phase 2 | 120 | No | H101 | Intra-tumor Injection of Oncolytic Viruses H101 Combined With or Without Radiotherapy in Refractory/Recurrent Gynecological Malignancies | Not published | NCT05051696 |
| Intravenous Injection of Oncolytic Virus Injection (RT-01) in Patients With Relapsed or Refractory T-cell Lymphoma | T-cell Lymphoma Virus Intravenous Injection | Biological: Oncolytic Virus Injection(RT-01) | 18 | Phase 1 | 6 | No | H101 | Intra-tumor Injection of Oncolytic Viruses H101 Combined With or Without Radiotherapy in Refractory/Recurrent Gynecological Malignancies | Not published | NCT05387226 |
| Oncolytic Virus Plus PD-1 Inhibitor to Patients With Advanced Pancreatic Cancer (PTCA199-8) | Pancreatic Cancer | Drug: H101 Drug: Camrelizumab | 18 - 80 | Phase 2 | 30 | No | H101  Camrelizumab | Oncolytic Virus Plus PD-1 Inhibitor to Patients With Advanced Pancreatic Cancer | Published:  The purpose of this study is to evaluate the efficacy of oncolytic virus plus PD-1 inhibitor to Patients with Advanced Pancreatic Cancer. | NCT06196671 |
| Dexamethasone, Carfilzomib, & Nivolumab With Pelareorep for Relapsed/Refractory Multiple Myeloma | Recurrent Plasma Cell Myeloma | Drug: Carfilzomib Drug: Dexamethasone Biological: Nivolumab Biological: Pelareorep | 18 | Phase 1 | 23 | No | Carfilzomib  Dexamethasone  Nivolumab  Pelareorep | PD1 Blockade and Oncolytic Virus in Relapsed Multiple Myeloma | Not published | NCT03605719 |
| OH2 Oncolytic Viral Therapy in Solid Tumors | Solid Tumor Gastrointestinal Cancer | Biological: OH2 injection, with or without irinotecan or HX008 | 18 - 75 | Phase 1 and 2 | 300 | No | OH2 injection, with or without irinotecan or HX008 | Phase I/II Study of OH2 Injection, an Oncolytic Type 2 Herpes Simplex Virus Expressing Granulocyte Macrophage Colony-Stimulating Factor, in Malignant Solid Tumors | Published: This phase I/II study evaluates the safety and efficacy of OH2 as single agent or in combination with HX008, an anti-PD-1 antibody, in patients with malignant solid tumors (gastrointestinal cancers, head and neck cancers, soft tissue sarcomas). OH2 is an oncolytic virus developed upon genetic modifications of the herpes simplex virus type 2 strain HG52, allowing the virus to selectively replicate in tumors. Meanwhile, the delivery of the gene encoding human granulocyte macrophage colony-stimulating factor (GM-CSF) may induce a more potent antitumor immune response. | NCT03866525 |
| OH2 Oncolytic Viral Therapy in Advanced Bladder Cancer | Advanced Bladder Carcinoma | Biological: OH2 injection | 18 - 75 | Phase 2 | 45 | No | OH2 injection | Efficacy and Safety Study of Oncolytic Virus (OH2) Intratumoral Injection in Locally Advanced or Metastatic Bladder Cancer a Phase Ⅱ Clinical Trial | Not published | NCT05248789 |
| OH2 Oncolytic Viral Therapy in Central Nervous System Tumors | Central Nervous System Tumors | Biological: OH2 injection | 18 | Phase 1 and 2 | 28 | No | OH2 injection | A Clinical Study of Oncolytic Virus (OH2) Injection in the Treatment of Patients Undergoing Surgery After Recurrence of Central Nervous System Tumors | Published: In the first phase, it mainly explores the safety, tolerability and preliminary effectiveness of two doses of OH2 injection in the treatment of patients with recurrent central nervous system tumors; to evaluate the biodistribution and virus shedding of OH2 injection administered in the tumor cavity; to evaluate the level of anti-HSV2 antibody in patients when OH2 injection is administered intracavitary to tumor; to determine the phase II recommended dose (RP2D) of OH2 injection in the treatment of recurrent glioblastoma. Phase IIa, to evaluate the preliminary efficacy of OH2 injection in the treatment of patients with recurrent glioblastoma after surgery, and to further evaluate the safety of OH2 in the treatment of relapsed glioblastoma. | NCT05235074 |
| Oncolytic Virus (OVV-01) Injection in the Treatment of Patients With Advanced Solid Tumors | Neoplasms | Drug: oncolytic virus (OVV-01) injection | 18 - 70 | Phase 1 | 50 | No | oncolytic virus (OVV-01) injection | A Single-arm, Open-label Clinical Study to Evaluate the Safety, Tolerability and Preliminary Efficacy of Oncolytic Virus (OVV-01) Injection Combined With or Without Immune Checkpoint Inhibitors in the Treatment of Patients With Advanced Solid Tumors | Published: Phase Ia: To investigate the safety, tolerability and efficacy of OVV-01 injection in the treatment of patients with advanced solid tumors (OVV-01 single dose gradient exploration).Phase Ib: To evaluate the safety, tolerability and efficacy of OVV-01 injection combined with immune checkpoint inhibitors pembrolizumab (anti-PD-1 monoclonal antibody) or atezolizumab (anti-PD-L1 monoclonal antibody) in the treatment of patients with advanced solid tumors (OVV-01 combined with PD-1/PD-L1 monoclonal antibody dose gradient exploration);Phase Ic: A cohort expansion of Phase Ib to further analyze the efficacy and safety of OVV-01 injection combined with immune checkpoint inhibitor injection in the treatment of advanced solid tumors. | NCT04787003 |
| SBRT and Oncolytic Virus Therapy Before Pembrolizumab for Metastatic TNBC and NSCLC (STOMP) | Metastatic Non-small Cell Lung Cancer Metastatic Triple-negative Breast Cancer | Biological: ADV/HSV-tk Drug: Valacyclovir Radiation: SBRT Drug: Pembrolizumab | 18 | Phase 2 | 57 | Yes | ADV/HSV-tk  Valacyclovir   SBRT  Pembrolizumab | Phase II Window of Opportunity Trial of Stereotactic Body Radiation Therapy and In Situ Oncolytic Virus Therapy in Metastatic Triple Negative Breast Cancer and Metastatic Non-Small Cell Lung Cancer Followed by Pembrolizumab | Not published | NCT03004183 |
| A Study of Recombinant Oncolytic Virus M1(VRT106) in Patients with Solid Tumors | Solid Tumor | Biological: VRT106 | 18 - 75 | Phase 1 | 30 | No | VRT106 | A Phase I Clinical Study to Evaluate the Safety, Tolerability, Biodistribution Characteristics, Biological Effects and Initial Efficacy of Recombinant Oncolytic Virus M1 for Injection (VRT106) in the Treatment of Patients With Locally Advanced or Metastatic Solid Tumors | Not published | [NCT06368921](https://clinicaltrials.gov/ct2/show/NCT06368921) |
| A Phase I Clinical Study of Intratumoral Injection Oncolytic Vaccinia Virus GC001 in Patient With Advanced Solid Tumors | Sarcoma Cervical Cancer Colon Cancer Lung Cancer Ovarian Cancer Pancreatic Cancer Hepatocellular Carcinoma Breast Cancer Gastric Cancer | Biological: A Phase I Clinical Study of Intratumoral Injection Oncolytic Vaccinia Virus GC001 in Patients With Advanced Solid Tumors | 18 - 75 | Phase 1 | 21 | No | A Phase I Clinical Study of Intratumoral Injection Oncolytic Vaccinia Virus GC001 in Patients With Advanced Solid Tumors | A Phase I Study Evaluating the Safety, Tolerability, Biodistribution and Shedding of the Virus, Pharmacodynamics, Immunogenicity, and Antitumor Activity of GC001 Oncolytic Vaccinia Virus Injection in Patient With Advanced Solid Tumors. | Not published | NCT06508307 |
| Safety of Recombinant Human IL-21-expressing Oncolytic Vaccinia Virus Injection (hV01) in Advanced Tumors | Advanced Solid Tumors | Biological: Recombinant human IL-21-expressing oncolytic vaccinia virus injection | 18 -75 | Phase 1 | 24 | No | Recombinant human IL-21-expressing oncolytic vaccinia virus injection | A Phase I Dose Escalation Study to Evaluate the Safety, Tolerance, Pharmacokinetics, and Biological Properties of Recombinant Human IL-21-expressing Oncolytic Vaccinia Virus Injection (hV01) in Patients With Advanced Malignant Solid Tumors | Not published | NCT05914376 |
| Evaluate Efficacy, Immunological Response of Intratumoral/?Intralesional Oncolytic Virus (OBP-301) in Metastatic Melanoma | Melanoma Stage III Melanoma Stage Iv | Drug: OBP-301 | 18 | Phase 2 | 4 | No | OBP-301 | Open-label, Multi-center Phase IIa Study to Evaluate the Efficacy, Safety, and Immunological Response of OBP-301, Telomerase Specific Replication-competent Oncolytic Adenovirus in Patients With Unresectable Metastatic Melanoma | Not published | NCT03190824 |
| TNFα and IL-2 Coding Oncolytic Adenovirus TILT-123 Monotherapy (TUNIMO) | Solid Tumor | Biological: TILT-123 | 18 | Phase 1 | 18 | No | TILT-123 | A Phase 1, Open-Label, Dose-escalation Clinical Trial of Tumor Necrosis Factor Alpha and Interleukin-2 Coding Oncolytic Adenovirus (TILT-123) in Patients With Injectable Solid Tumors | Published: This is an open-label, phase 1, dose-escalation, multicenter trial evaluating the safety of oncolytic adenovirus TILT-123 as monotherapy in advanced solid tumor patients. | NCT04695327 |
| A Study of an Intratumoral Oncolytic Virus in Patients With Advanced Metastatic Solid Tumors | Metastatic Cancer Solid Tumors Advanced Cancer | Biological: ASP9801 Combination Product: Pembrolizumab | 18 | Phase 1 | 72 | No | ASP9801  Pembrolizumab | A Phase 1, Open-label Study of ASP9801, an Oncolytic Virus, Administered by Intratumoral Injection as a Single Agent and in Combination With Pembrolizumab in Subjects With Advanced/Metastatic Solid Tumors | Not published | NCT03954067 |
| A Clinical Study on Oncolytic Virus Injection (R130) for the Treatment of Advanced Solid Tumors | Sarcoma Carcinoma Breast Cancer Pancreatic Cancer Colorectal Cancer Gastric Cancer Liver Cancer Lung Cancer Gynecologic Cancer | Drug: Recombinant oncolytic herpes simplex virus type 1 (R130) | 18 - 75 | Early Phase 1 | 20 | No | Recombinant oncolytic herpes simplex virus type 1 (R130) | A Clinical Safety and Efficacy Study on Oncolytic Virus Injection (R130) for the Treatment of Advanced Solid Tumors | Not published | NCT05860374 |
| Oncolytic Virus Plus Anti-PD1 and Chemotherapy as Preoperative Therapy for Patients With BRPC/LAPC | Pancreatic Cancer | Drug: Oncolytic virus Plus Anti-PD1 and Chemotherapy | 18 - 75 | Phase 1 | 20 | No | Oncolytic virus Plus Anti-PD1 and Chemotherapy | Oncolytic Virus Plus Anti-PD1 and Chemotherapy as Preoperative Therapy for Patients With Borderline Resectable and Locally Advanced Pancreatic Cancer | Not published | NCT06346808 |
| Safety and Efficacy of the ONCOlytic VIRus Armed for Local Chemotherapy, TG6002/?5-FC, in Recurrent Glioblastoma Patients (ONCOVIRAC) | Glioblastoma Brain Cancer | Drug: Combination of TG6002 and 5-flucytosine (5-FC, Ancotil®) | 18 | Phase 1 and 2 | 78 | No | Combination of TG6002 and 5-flucytosine (5-FC, Ancotil®) | Safety and Efficacy of the ONCOlytic VIRus Armed for Local Chemotherapy, TG6002/5-FC, in Recurrent Glioblastoma Patients | Not published | NCT03294486 |
| OH2 Oncolytic Viral Therapy in Non-Muscle-Invasive Bladder Cancer | Non-muscle-invasive Bladder Cancer | Biological: OH2 injection | 18 - 80 | Phase 1 and 2 | 30 | No | OH2 injection | Oncolytic Virus (OH2) Adjuvant Therapy After Transurethral Resection of Bladder Tumor in Non-Muscle-Invasive Bladder Cancer Who Have Failed First-line Prophylactic Intravesical Instillation Therapy: a Phase Ⅰb/Ⅱ Clinical Trial | Not published | NCT05232136 |
| Safety Study of GL-ONC1, an Oncolytic Virus, in Patients With Advanced Solid Tumors | Advanced Cancers (Solid Tumors) | Biological: GL-ONC1 | 18 | Phase 1 | 43 | No | GL-O GL-ONC1NC1 | Phase I Study of the Safety, Tolerability,and Tumor-Specific Replication of the Intravenous Administration of Green Fluorescent Protein Encoded Genetically Engineered Attenuated Vaccinia Virus, GL-ONC1, in Patients With Advanced Solid Organ Cancers. | Not published | NCT00794131 |
| A Clinical Study on Oncolytic Virus Injection (R130) for the Treatment of Relapsed/?Refractory Ovarian Cancer | Ovarian Cancer Peritoneal Carcinomatosis Fallopian Tube Cancer | Drug: Recombinant oncolytic herpes simplex virus type 1 (R130) | 18 - 75 | Early Phase 1 | 10 | No | Recombinant oncolytic herpes simplex virus type 1 (R130) | A Clinical Safety and Efficacy Study on Oncolytic Virus Injection (R130) for the Treatment of Relapsed/Refractory Ovarian Cancer | Not published | NCT05801783 |
| A Clinical Study on Oncolytic Virus Injection (R130 OV) for the Treatment of Advanced Solid Tumors | Sarcoma Carcinoma Digestive Cancer Breast Cancer Lung Cancer Brain Cancer Melanoma Gynecologic Cancer Head and Neck Cancer Kidney Cancer | Drug: Recombinant oncolytic herpes simplex virus type 1 (R130) | 18 - 75 | Early Phase 1 | 20 | No | Recombinant oncolytic herpes simplex virus type 1 (R130) | An Open, Single-armed, Clinical Safety and Efficacy Study on Oncolytic Virus Injection (R130 OV) for the Treatment of Advanced Solid Tumors | Not published | NCT05961111 |
| A Study of Intratumoral Administration of Oncolytic Virus Injection (RT-01) in Patients With Advanced Solid Tumors | Advanced Solid Tumor | Biological: Oncolytic Virus Injection(RT-01) | 18 | Phase 1 | 7 | No | Oncolytic Virus Injection(RT-01) | A Single-Arm, Open-Label, Dose Escalation Study to Evaluate Safety and Efficacy of Intratumoral Injection of Oncolytic Virus Injection (RT-01) in Patients With Advanced Solid Tumors | Not published | NCT05205408 |
| A Study of GL-ONC1, an Oncolytic Vaccinia Virus, in Patients With Advanced Peritoneal Carcinomatosis | Peritoneal Carcinomatosis | Biological: GL-ONC1 | 18 | Phase 1 and 2 | 9 | No | GL-ONC1 | Phase I/II Study of Intraperitoneal Administration of GL-ONC1, a Genetically Modified Vaccinia Virus, in Patients With Peritoneal Carcinomatosis | Published: The purpose of this study is to determine whether GL-ONC1, an attenuated vaccinia virus, is safe when administered to patients with peritoneal carcinomatosis via an infusion within the abdominal cavity through an implanted catheter. The study seeks also to arrive at a recommended dose and schedule for future investigations, evidence of anti-tumor activity, detection of virus in body fluids, analysis of viral delivery to tumor and normal cells, and to evaluate if there is an antibody response to vaccinia virus. | NCT01443260 |
| A Study of Oncolytic Virus Injection (RT-01) in Patients With Extensive-Stage Small Cell Lung Cancer | Advanced Solid Tumor | Biological: Oncolytic Virus Injection(RT-01) | 18 | Phase 1 | 20 | No | Oncolytic Virus Injection(RT-01) | A Single-Arm, Open-Label, Exploratory Study to Evaluate Safety and Efficacy of Oncolytic Virus Injection (RT-01) in Patients With Extensive-Stage Small Cell Lung Cancer | Not published | NCT05205421 |
| Clinical Study on the Safety and Efficacy of Novel Oncolytic Virus in the Treatment of Recurrent Malignant Glioma | Glioblastoma Multiforme Gliomas, Malignant | Biological: ON-01 Drug: ONF | 18 - 65 | Phase 1 and 2 | 38 | No | ON-01  ONF | Clinical Study on the Safety and Efficacy of Novel Oncolytic Virus in the Treatment of Recurrent Malignant Glioma | Not published | NCT06562621 |
| A Clinical Study on Oncolytic Virus Injection (R130) for the Treatment of Relapsed/Refractory Advanced Solid Tumors | Lung Cancer Bronchial Cancer Non Small Cell Lung Cancer Small Cell Lung Cancer Sarcoma Colorectal Cancer Gastric Cancer Liver Cancer Breast Cancer Pancreatic Cancer Head and Neck Cancer Ovarian Cancer | Drug: Recombinant oncolytic herpes simplex virus type 1 (R130) | 18 - 75 | Early Phase 1 | 24 | No | Recombinant oncolytic herpes simplex virus type 1 (R130) | A Clinical Safety and Efficacy Study on Oncolytic Virus Injection (R130) for the Treatment of Relapsed/Refractory Advanced Solid Tumors | Not published | NCT05886075 |
| Study of Oncolytic Virus in Combination With HX-008 and Axitinib in Melanoma Patients With Liver Metastasis | Melanoma Stage IV | Drug: Recombinant Oncolytic HSV-2 Therapeutic Injecta (Vero Cell) for Human Use (rHSV2hGM-CSF) Drug: Recombinant humanized anti-PD-1 monoclonal antibody for injection Drug: Axitinib | 18 - 75 | Phase 1 | 30 | No | Recombinant Oncolytic HSV-2 Therapeutic Injecta (Vero Cell) for Human Use (rHSV2hGM-CSF)  Recombinant humanized anti-PD-1 monoclonal antibody for injection  Axitinib | Safety and Efficacy Study of Oncolytic Virus（Intratumoral Injection）in Combination With HX-008（Intravenous Injection）and Axitinib in Melanoma Patients With Liver Metastasis Who Lack or Become Refractory to Standard Treatment | Not published | NCT05070221 |
| Study of Oncolytic Virus in Combination With HX-008 and Radiotherapy in Melanoma Patients With Liver Metastasis | Melanoma Stage IV | Drug: Recombinant Oncolytic HSV-2 Therapeutic Injecta (Vero Cell) for Human Use (rHSV2hGM-CSF) Drug: Recombinant humanized anti-PD-1 monoclonal antibody for injection Radiation: RT | 18 - 75 | Phase 1 | 15 | No | Recombinant Oncolytic HSV-2 Therapeutic Injecta (Vero Cell) for Human Use (rHSV2hGM-CSF)  Recombinant humanized anti-PD-1 monoclonal antibody for injection  RT | Safety and Efficacy Study of Oncolytic Virus（Intratumoral Injection）in Combination With HX-008（Intravenous Injection）and Radiotherapy for Liver Metastasis in Melanoma Patients With Liver Metastasis Who Lack or Become Refractory to Standard Treatment | Not published | NCT05068453 |
| A Clinical Study on Oncolytic Virus Injection (R130) for the Treatment of Advanced Bone and Soft Tissue Tumors | Osteosarcoma Sarcoma Soft Tissue Sarcoma Bone Tumor | Drug: Recombinant oncolytic herpes simplex virus type (R130) | 16 - 75 | Early Phase 1 | 9 | No | Recombinant oncolytic herpes simplex virus type Ⅰ (R130) | A Clinical Safety and Efficacy Study on Oncolytic Virus Injection (R130) for the Treatment of Advanced Bone and Soft Tissue Tumors | Not published | NCT06171282 |
| Safety and Efficacy of CG0070 Oncolytic Virus Regimen for High Grade NMIBC After BCG Failure (BOND2) | Bladder Cancer | Biological: CG0070 | 18 | Phase 2 | 66 | yes | CG0070 | An Open Label, Single Arm, Phase II, Multicenter Study of the Safety and Efficacy of CG0070 Oncolytic Vector Regimen in Patients With Non-Muscle Invasive Bladder Carcinoma Who Have Failed BCG (Bacillus Calmette-Guerin) Therapy and Refused Cystectomy | Not published | NCT02365818 |
| A Clinical Study on Oncolytic Virus Injection (R130) for the Treatment of Relapsed/?Refractory Cervical and Endometrial Cancer | Cervical Cancer  Endometrial Cancer  Advanced Cancer | Drug: Recombinant oncolytic herpes simplex virus type ? (R130) | 18 - 75 | Early Phase 1 | 20 | No | Recombinant oncolytic herpes simplex virus type Ⅰ (R130) | A Clinical Safety and Efficacy Study on Oncolytic Virus Injection (R130) for the Treatment of Relapsed/Refractory Cervical and Endometrial Cancer | Not published | NCT05812677 |
| Exploratory Study of a Novel Oncolytic Vaccinia Virus RGV004 in the Treatment of Refractory/Relapsed B-cell Lymphoma | Relapsed or Refractory B-cell Lymphoma | Biological: RGV004 | 18 - 75 | Phase 1 | 25 | No | RGV004 | Exploratory Study of a Novel Oncolytic Vaccinia Virus Expressing Bispecific Antibody in the Treatment of Refractory/Relapsed B-cell Lymphoma | Not published | NCT04887025 |
| A Clinical Study on Oncolytic Virus Injection (R130 OV) for the Treatment of Relapsed/Refractory Head and Neck Cancer | Head and Neck Cancer Esophageal Cancer Otorhinolaryngologic Neoplasms Ear Cancer Nose Cancer Laryngeal Cancer Pharyngeal Cancer | Drug: Recombinant oncolytic herpes simplex virus type 1 (R130) | 18 - 75 | Early Phase 1 | 9 | No | Recombinant oncolytic herpes simplex virus type 1 (R130) | A Clinical Safety and Efficacy Study on Oncolytic Virus Injection (R130) for the Treatment of Relapsed/Refractory Head and Neck Cancer | Not published | NCT05830240 |
| A Phase I/?II Study of Pexa-Vec Oncolytic Virus in Combination With Immune Checkpoint Inhibition in Refractory Colorectal Cancer | Colorectal CancerColorectal CarcinomaColorectal AdenocarcinomaRefractory CancerColorectal Neoplasms | Drug: Durvalumab Drug: Tremelimumab Biological: Pexa-Vec Biological: Pexa-Vec | 18 | Phase 1 and 2 | 34 | Yes | Durvalumab  Tremelimumab  Pexa-Vec | A Phase I/II Study of Pexa-Vec Oncolytic Virus in Combination With Immune Checkpoint Inhibition in Refractory Colorectal Cancer | Published: To determine the safety, tolerability and feasibility of Pexa-Vec oncolytic virus in combination with immune checkpoint inhibition in patients with refractory metastatic colorectal cancer. | NCT03206073 |
| A Study of Oncolytic Virus Injection (RT-01) in Combination With PD-1 Inhibitor in Patients With Advanced Solid Tumors | Advanced Solid Tumor | Biological: Oncolytic Virus Injection(RT-01) | 18 | Phase 1 | 50 | No | Oncolytic Virus Injection(RT-01) | A Single-Arm, Open-Label, Exploratory Study to Evaluate Safety and Efficacy of Oncolytic Virus Injection (RT-01) in Combination With PD-1 Inhibitor (Nivolumab) in Patients With Advanced Solid Tumors | Not published | NCT05228119 |
| A Clinical Study on Oncolytic Virus Injection (R130) for the Treatment of Relapsed/?Refractory Bone and Soft Tissue Tumors | OsteosarcomaSarcomaSarcoma,Soft TissueBone Tumor | Drug: Recombinant oncolytic herpes simplex virus type ? (R130) | 14 - 75 | Early Phase 1 | 20 | No | Recombinant oncolytic herpes simplex virus type Ⅰ (R130) | A Clinical Safety and Efficacy Study on Oncolytic Virus Injection (R130) for the Treatment of Relapsed/Refractory Bone and Soft Tissue Tumors | Not published | NCT05851456 |
| MEM-288 Oncolytic Virus Alone and in Combination With Nivolumab in Solid Tumors Including Non-Small Cell Lung Cancer | Solid TumorAdvanced CancerMetastatic CancerNon Small Cell Lung CancerCutaneous Squamous Cell CarcinomaMerkel Cell CarcinomaMelanomaPancreatic CancerTriple Negative Breast CancerHead and Neck Cancer | Biological: MEM-288 Intratumoral Injection Biological: Nivolumab | 18 | Phase 1 | 61 | No | MEM-288 Intratumoral Injection  Nivolumab  Docetaxel | Phase I Study Evaluating MEM-288 Oncolytic Virus Alone and in Combination With Standard of Care Therapy in Advanced Solid Tumors | Not published | NCT05961111 |
| Study to Assess the Safety and Preliminary Efficacy of STI-1386 Oncolytic Virus in Relapsed or Refractory Solid Tumors | CancerCancer of PancreasSarcomaHepatic MetastasisSolid Tumor | Drug: STI-1386 | 18 | Phase 1 | 36 | No | STI-1386 | A Phase 1b, Dose-Escalation Study of the Safety and Preliminary Efficacy of STI-1386 Oncolytic Virus in Patients With Relapsed or Refractory Solid Tumors | Not published | NCT05361954 |
| Oncolytic Virus H101 Combined With Lenvatinib Plus Toripalimab Compared With FOLFOX in Patients With Advanced Biliary Tract Cancer (OPTIONS-06) | Biliary Tract Cancer (BTC) | Drug: TORIPALIMAB INJECTION(JS001 ) Drug: Lenvatinib Drug: H101 Drug: FOLFOX | 18 | Phase 2 | 74 | No | TORIPALIMAB INJECTION(JS001 )  Lenvatinib  H101  FOLFOX | Oncolytic Virus H101 Combined With Lenvatinib Plus Toripalimab Compared With FOLFOX in Patients With Advanced Biliary Tract Cancer (OPTIONS-06): a Multicenter, Randomized, Phase 2 Study | Not published | NCT06919848 |
| UARK 2014-21 A Phase II Trial of Oncolytic Virotherapy by Systemic Administration of Edmonston Strain of Measles Virus | Multiple Myeloma | Drug: MV-NIS | 18 - 75 | Phase 2 | 2 | Yes | MV-NIS | A Phase II Trial of Oncolytic Virotherapy by Systemic Administration of Edmonston Strain of Measles Virus, Genetically Engineered to Express NIS, With Cyclophosphamide, in Patients With Recurrent of Refractory Multiple Myeloma | Not published | NCT02192775 |
| Recombinant Measles Virus Vaccine Therapy and Oncolytic Virus Therapy in Treating Patients With Progressive, Recurrent, or Refractory Ovarian Epithelial Cancer or Primary Peritoneal Cancer | Ovarian CancerPrimary Peritoneal Cavity Cancer | Biological: carcinoembryonic antigen-expressing measles virus Biological: oncolytic measles virus encoding thyroidal sodium iodide symporter Genetic: reverse transcriptase-polymerase chain reaction Other: laboratory biomarker analysis | 18 - 120 | Phase 1 | 37 | Yes | carcinoembryonic antigen-expressing measles virus  oncolytic measles virus encoding thyroidal sodium iodide symporter  reverse transcriptase-polymerase chain reaction  laboratory biomarker analysis | Phase I Trial of Intraperitoneal Administration of a) a CEA-Expressing Derivative, and b) a NIS-Expressing Derivative Manufactured From a Genetically Engineered Strain of Measles Virus in Patients With Recurrent Ovarian Cancer | Not published | NCT00408590 |
| GL-ONC1 Oncolytic Immunotherapy in Patients With Recurrent or Refractory Ovarian Cancer | Ovarian CancerPeritoneal CarcinomatosisFallopian Tube Cancer | Biological: GL-ONC1 alone, or in combination with chemotherapy with or without bevacizumab | 21 | Phase 1 and 2 | 46 | No | GL-ONC1 alone, or in combination with chemotherapy with or without bevacizumab | Phase 1b & 2 Study With GL-ONC1 Oncolytic Immunotherapy in Patients With Recurrent or Refractory Ovarian Cancer (VIRO-15) | Published: The purpose of this study is to determine if GL-ONC1 oncolytic immunotherapy is well tolerated with anti-tumor activity in patients diagnosed with recurrent or refractory ovarian cancer and peritoneal carcinomatosis. | NCT02759588 |
| TNFalpha and Interleukin 2 Coding Oncolytic Adenovirus TILT-123 During TIL Treatment of Advanced Melanoma (TUNINTIL) | Metastatic Melanoma | Biological: TILT-123 | 18 - 75 | Phase 1 | 17 | No | TILT-123  TIL  Cyclophosphamide  Fludarabine | A Phase 1, Open-label, Single-arm Study to Investigate the Safety, Tolerability, and Preliminary Efficacy of TNFa and IL-2 Coding Oncolytic Adenovirus TILT-123 in Combination With Lymphocyte-depleting Chemotherapy and Tumor-infiltrating Lymphocytes in Melanoma Patients. | Not published | NCT06961786 |
| Safety and Efficacy of Recombinant Oncolytic Adenovirus L-IFN Injection in Relapsed/?Refractory Solid Tumors Clinical Study (YSCH-01) | Head and Neck CancerMelanomaBreast CancerBladder CancerOvarian CarcinomaCervical CarcinomaLung Cancer | Drug: Recombinant L-IFN adenovirus injection | 18 - 75 | Early Phase 1 | 28 | No | Recombinant L-IFN adenovirus injection | Safety and Efficacy of Recombinant L-IFN Adenovirus Injection in Relapsed/Refractory Solid Tumors: a Single/Multicenter, Dose-increasing, Cohort Extension Clinical Study | Published: This is an open-label, dose escalation study of the safety and tolerability of Recombinant oncolytic adenovirus L-IFN injection（YSCH-01） when administered via intratumoral injection in patients with advanced solid tumors. The purpose of this study is to assess the safety and tolerability of Recombinant L-IFN adenovirus injectionand to determine the recommended phase 1 dose for further study. The study will also evaluate antitumor activity, objective response rate, pharmacokinetics and virus shedding of Recombinant L-IFN adenovirus injection | NCT05180851 |
| "neoBREASTIM": Atezolizumab Plus RP1 Oncolytic Immunotherapy in the NeoAdjuvant Setting of Triple-Negative Breast Cancer (neoBREASTIM) | Triple Negative Breast Neoplasms | Combination Product: Atezolizumab + RP1 | 18 | Phase 1 and 2 | 51 | No | Atezolizumab + RP1 | "neoBREASTIM": A Phase 2 Study of Atezolizumab Plus RP1 Oncolytic Immunotherapy in the NeoAdjuvant Setting of Triple-Negative Breast Cancer (TNBC) | Not published | [NCT06067061](https://cdek.pharmacy.purdue.edu/trial/NCT06067061/) |
| First in Human Study of NG-350A (an Oncolytic Adenoviral Vector Which Expresses an Anti-CD40 Antibody) (FORTITUDE) | Metastatic CancerEpithelial Tumor | Biological: NG-350A | 18 | Phase 1 | 28 | Yes | NG-350A | A Multicentre, Open Label, Non-randomised First in Human Study of NG-350A (Monotherapy), and NG-350A With a Check Point Inhibitor in Patients With Metastatic or Advanced Epithelial Tumours | Published: This study will evaluate the safety, tolerability and preliminary efficacy and also pharmacokinetics, immunogenicity and other pharmacodynamic effects to elucidate the mechanism of action of NG-350A, either alone or in combination with a check point inhibitor, in patients with advanced or metastatic epithelial tumours. | NCT03852511 |
| Oncolytic Adenovirus(H101) Combined With PD-1 Inhibitors in Patients With Advanced Malignant Pleural Mesothelioma | Malignant Pleural Mesothelioma, Advanced | Drug: Oncolytic Adenovirus H101 Drug: Programmed death receptor-1 inhibitor | 18 - 75 |  | 15 | No | Oncolytic Adenovirus H101  Programmed death receptor-1 inhibitor | Observation of the Efficacy and Safety of Oncolytic Adenovirus Injection Combined With Programmed Death Receptor Inhibitors in Treatment of Advanced Malignant Pleural Mesothelioma : a Single Center, Prospective, Case Registration Study | Not published | NCT06031636 |
| A Study of CF33-hNIS (VAXINIA), an Oncolytic Virus, as Monotherapy or in Combination With Pembrolizumab in Adults With Metastatic or Advanced Solid Tumors (MAST) | Solid Tumor Solid Carcinoma Solid Tumor, Adult Metastatic Cancer Advanced Solid Tumor Cholangiocarcinoma Bile Duct Cancer | Biological: CF33-hNIS Biological: Pembrolizumab | 18 | Phase 1 | 100 | No | CF33-hNIS  Pembrolizumab  Modified FOLFOX | A Phase I, Dose Escalation Safety and Tolerability Study of VAXINIA (CF33-hNIS), Administered Intratumorally or Intravenously as a Monotherapy or in Combination With Pembrolizumab in Adult Patients With Metastatic or Advanced Solid Tumors (MAST). | Not published | NCT05346484 |
| Oncolytic Adenovirus Combined With PD-1 Inhibitor in Patients With Non-muscle-invasive Bladder Cancer | Bladder Cancer | Drug: H101, Camrelizumab | 18 - 80 | Phase 2 | 25 | No | H101, Camrelizumab | Phase II Single Center Open-Label Single-Arm Study of the Safety and Efficacy of Oncolytic Adenovirus H101 Combined With PD-1 Inhibitor in Patients With Non-muscle-invasive Bladder Cancer Who Failed BCG Therapy | Not published | NCT05564897 |
| Oncolytic Adenovirus TILT-123 and Avelumab for Treatment of Solid Tumors Refractory to or Progressing After Anti-PD(L)1 (AVENTIL) | MelanomaHead and Neck Squamous Cell Carcinoma | Biological: TILT-123 Drug: Avelumab | 18 | Phase 1 | 15 | No | TILT-123  Avelumab | A Phase I Open-Label, Dose-escalation Clinical Trial of Tumor Necrosis Factor Alpha and IL-2 Coding Oncolytic Adenovirus TILT-123 and Avelumab in Solid Tumor Patients (Melanoma and SCCHN) Refractory to or Progressing After Anti-PD(L)1 | Not published | NCT05222932 |
| Phase I Endovenous Administration of Oncolytic Adenovirus ICOVIR-5 in Patients With Advanced or Metastatic Melanoma | Locally Advanced or Metastatic Melanoma | Biological: ICOVIR-5 | 18 | Phase 1 | 14 | No | ICOVIR-5 | Phase I Clinical Trial of Endovenous Administration of Conditionally Replicative Adenovirus ICOVIR-5 in Patients With Locally Advanced or Metastatic Melanoma | Published: The investigators will evaluate the safety of a single endovenous infusion of ICOVIR5 in adults with locally advanced and metastatic melanoma. ICOVIR5 consists in a conditionally replicative or oncolytic adenovirus. | NCT01864759 |
